# MAP2K6 reprograms NAD^+^ metabolism via the HMGA1–NAMPT axis to sensitize ovarian cancer cells to PARP inhibition

**DOI:** 10.1038/s41419-026-09104-2

**Published:** 2026-07-17

**Authors:** Ming Wen, Zhuohang Chen, Dongdong Zhan, Mingwei Liu, Shuran Tan, Fang Zhu, Siyu Yang, Jinru Xie, Yu Zhang, Jianjian Li, Lunquan Sun, Rong Tan

**Affiliations:** 1 Department of Oncology, Xiangya Cancer Center, Xiangya Hospital, Central South University, Changsha, China; 2 Key Laboratory of Molecular Radiation Oncology Hunan Province, Changsha, China; 3 National Clinical Research Center for Geriatric Disorders, Xiangya Hospital, Central South University, Changsha, China; 4 State Key Laboratory of Proteomics, Beijing Proteome Research Center, National Center for Protein Sciences (Beijing), Beijing Institute of Lifeomics, Beijing, China; 5 Beijing Pineal Diagnostics Co., Ltd., Beijing, China; 6 Department of Gynecology, Xiangya Hospital, Central South University, Changsha, Hunan China; 7 Gynecological Oncology Research and Engineering Center of Hunan Province, Changsha, Hunan China; 8 Hunan International Science and Technology Collaboration Base of Precision Medicine for Cancer, Changsha, China; 9 Center for Molecular Imaging of Central South University, Xiangya Hospital, Changsha, China; 10 Hunan key laboratory of aging biology, Xiangya Hospital, Central South University, Changsha, China

**Keywords:** Predictive markers, Ovarian cancer, Targeted therapies, Stress signalling

## Abstract

PARP inhibitors (PARPi) selectively target cancers with homologous recombination deficiency (HRD), yet emerging evidence suggests that additional determinants beyond BRCA/HRD status modulate PARPi response. Here, by integrating unbiased proteomic profiling with systematic drug-response phenotyping across 11 ovarian cancer cell lines, we identify MAP2K6 as a previously unrecognized regulator of PARP1 activation and an important determinant of PARP inhibitor sensitivity in BRCA-wild-type ovarian cancer. MAP2K6 promotes global PARP1-mediated PARylation and enhances cellular responsiveness to the PARP inhibitor olaparib both in vitro and in vivo. Mechanistically, MAP2K6 can phosphorylate and activate HMGA1, which in turn transcriptionally upregulates NAMPT, resulting in increased intracellular NAD^+^ levels, and sustained PARP1 activation. Genetic depletion of HMGA1 or NAMPT abolishes MAP2K6-driven PARylation and PARPi sensitization, whereas loss of MAP2K6 reduces NAD^+^ levels, attenuates PARP1 activation, and confers resistance to PARPi. We propose that MAP2K6 promotes PARP inhibitor sensitivity by coupling metabolic control of NAD^+^ supply to PARP1 activation, thereby potentially helping extend the therapeutic scope of PARP inhibition beyond homologous recombination-deficient ovarian cancer.

## Introduction

Poly(ADP-ribose) polymerase (PARP) inhibitors represent a class of targeted anticancer agents that exploit synthetic lethality to selectively eliminate tumors with defects in DNA repair [1, 2]. By inhibiting PARP1-dependent DNA damage signaling, these agents induce catastrophic genomic instability in cancers harboring BRCA1/2 mutations or broader homologous recombination deficiency (HRD). Olaparib and related PARP inhibitors were therefore initially developed and approved for HR-deficient malignancies. However, accumulating clinical evidence has revealed significant progression-free survival benefits in subsets of patients lacking canonical BRCA or HR alterations [3–6], indicating that PARPi efficacy extends beyond traditional HR-deficient contexts. These findings raise an important question of what additional molecular determinants regulate tumor responsiveness to PARPi therapy.

PARP1, the primary catalytic target of PARP inhibitors, functions as a DNA damage sensor that detects single- and double-strand breaks and catalyzes the synthesis of poly(ADP-ribose) (PAR) chains using NAD^+^ as a substrate [7–9]. PARylation coordinates the recruitment and spatial organization of DNA repair factors, chromatin remodelers, and replication stress regulators [10]. Beyond its role in DNA repair, PARP1 exhibits substantial basal activity that contributes to chromatin organization, transcriptional regulation, and genome surveillance [11, 12]. Importantly, PARP1 enzymatic output is tightly constrained by intracellular NAD^+^ availability, thereby linking PARP1 activity to cellular metabolic state [13]. Thus, the intrinsic functional state of PARP1-its activation level, chromatin engagement, and NAD^+^ dependence-may strongly influence PARPi efficacy, independent of HR capacity. Yet the upstream signaling and metabolic networks that tune PARP1 activity in tumors remain incompletely defined.

Cells adapt to genotoxic and metabolic stress through kinase signaling networks that coordinate DNA repair, transcription, and metabolism. Among these, the mitogen-activated protein kinase (MAPK) networks integrate environmental and intracellular stress cues through hierarchical phosphorylation cascades. Early studies have revealed intricate reciprocal crosstalk between MAPK signaling and PARP1 activity. Phosphorylated ERK2 can directly bind to PARP1 and stimulate PARP1 enzymatic activity in a DNA damage-independent manner, thereby linking mitogenic signaling to PARylation dynamics [14–16]. Conversely, PARP1 has been shown to modulate MAPK pathway output by repressing BRAF-X1 mRNA translation or promoting the nuclear translocation of p38 MAPK [17–19]. The dual-specificity kinase MAP2K6 (MKK6), a principal upstream activator of the p38 cascade, has been implicated in stress adaptation, DNA repair, and therapy resistance [20–22]. However, whether MAP2K6 directly regulates PARP1 function or influences tumor sensitivity to PARP inhibition remains unclear.

In this study, we combined unbiased proteomic profiling of ovarian cancer cell lines with quantitative PARPi IC50 measurements to identify regulators of PARPi sensitivity. We discovered that MAP2K6 acts as a previously unrecognized modulator of PARP1 activation and olaparib response. Mechanistically, MAP2K6 drives HMGA1-dependent transcriptional activation of NAMPT, boosting NAD^+^ biosynthesis and sustaining PARP1 hyperactivation and chromatin engagement. In this hyperactivated state, PARP1 becomes more susceptible to inhibitor-induced trapping, thereby amplifying olaparib cytotoxicity. Collectively, these findings define a MAP2K6-HMGA1-NAMPT signaling axis that links stress-kinase signaling to tumor NAD^+^ metabolism and PARP1 functional state, suggesting mechanistic framework that could inform extension of PARP-targeted therapy, pending further clinical validation.

## Results

### MAP2K6 expression predicts olaparib sensitivity in ovarian cancer

**Fig. 1:**
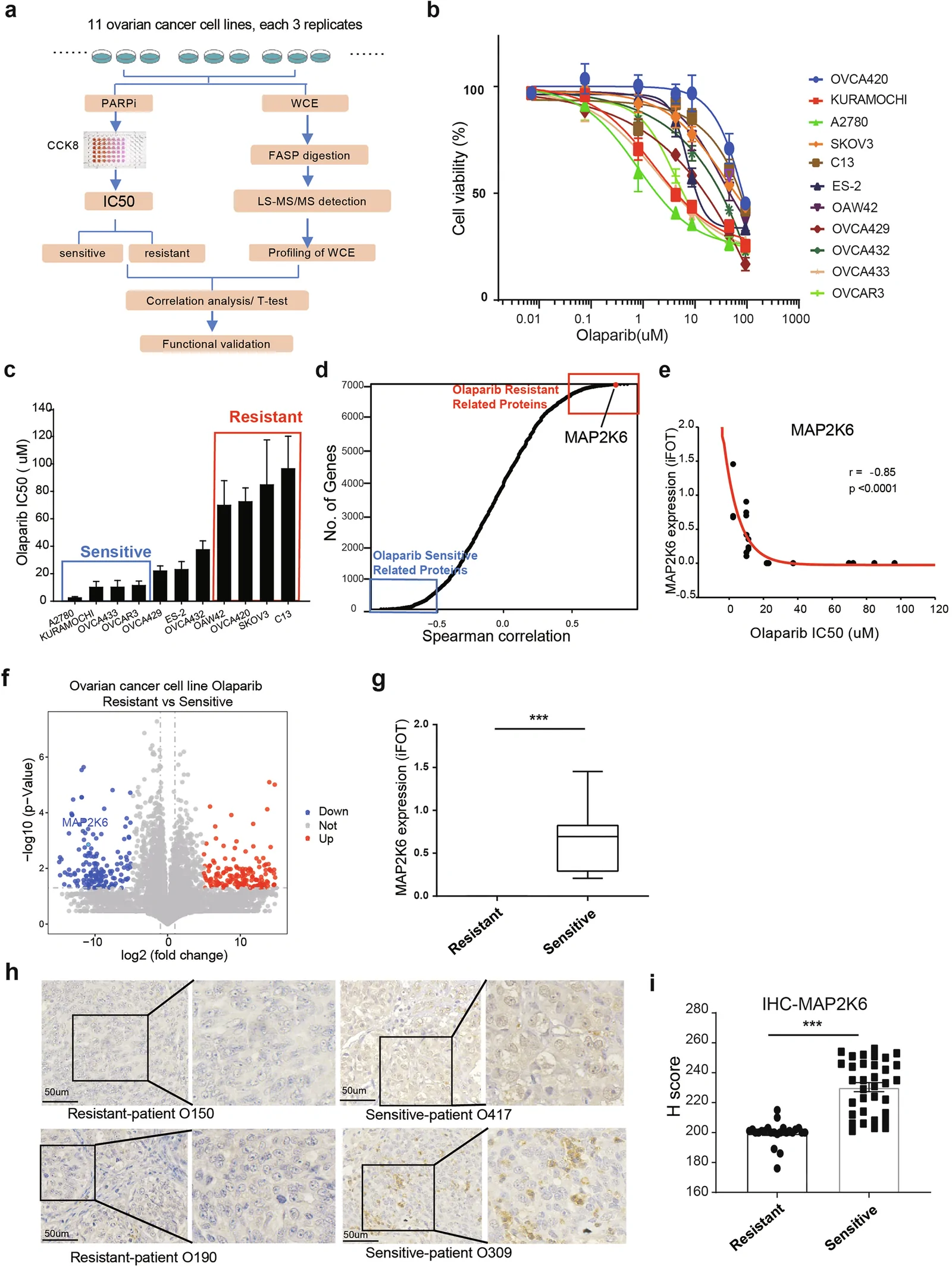
MAP2K6 is a predictive marker of PARP inhibitor sensitivity in ovarian cancer. **a** Workflow depicting the identification of PARPi-sensitive markers using MS-based quantitative proteomics, IC50 measurement of PARPi, and bioinformatic analyses across 11 ovarian cancer cell lines. Dose-response curves with IC50 values for olaparib in indicated 11 ovarian cancer cell lines (**b**). Cytotoxicity curves represent *n* = 3 experiments with 6 replicates per drug concentration for each experiment. Bar graphs (**c**) show quantification of olaparib IC50 values. Based on the sensitivity to olaparib, the cells were classified into two groups. (error bar= mean ± SEM, *n* = 3). **d** Spearman correlation analysis between protein abundance (iFOT) and olaparib IC50 values. Genes with Spearman correlation < −0.5 and *p* ≤ 0.05 were defined as olaparib-sensitive genes (red box), while genes with Spearman correlation > 0.5 and *p* ≤ 0.05 were defined as olaparib-resistant genes (blue box). **e** Spearman’s correlation analysis between MAP2K6 protein abundance and olaparib sensitivity. **f** Volcano plot showing differentially expressed genes identified between the olaparib-resistant and olaparib-sensitive cell groups. Red and blue indicate significantly up- or down-regulated genes (*p* < 0.05, log_2_ (FC) > 1); gray indicates nonsignificant changes. **g** MAP2K6 protein levels in olaparib-sensitive cells and olaparib-resistant cells. Statistical significance was determined using two-tailed unpaired Student’s *t* tests (***p < 0.001). Immunohistochemical staining of MAP2K6 in tumor samples from olaparib-resistant (*n* = 8) and -sensitive (*n* = 11) ovarian cancer patients. Representative images (**h**) and quantification (**i**) are shown. (two-tailed unpaired Student’s *t* tests, ***p < 0.001; error bar = mean ± SEM; each tumor quantified in three random fields; scale bars, 50 μm).

Increasing evidence indicates that PARP inhibitors can elicit therapeutic responses in a subset of ovarian cancers lacking BRCA mutations. To identify molecular determinants of this sensitivity, we assessed the response to olaparib across a panel of 11 ovarian cancer cell lines with varying genetic backgrounds and only KURAMOCHI cell got BRCA2 mutation [23] (Fig. 1a). The cell lines exhibited a broad range of olaparib sensitivity, as measured by half-maximal inhibitory concentration (IC50) values (Fig. 1b). A2780, KURAMOCHI, OVCA433 and OVCAR3 cells displayed high sensitivity, with IC50 values below 10 µM, whereas C13, SKOV3, OVCA420 and OAW42 cells were resistant, exhibiting IC50 values greater than 50 µM (Fig. 1c).

To uncover molecular features associated with differential olaparib sensitivity, we performed label-free quantitative proteomic profiling on all 11 cell lines (three biological replicates per line; Extended Data Fig. 1a). Across the dataset, 7802 proteins were quantified, with approximately 4500 identified per cell line (Extended Data Fig. 1b-c). Correlation analysis between protein abundance (iFOT values) and olaparib IC50 revealed MAP2K6 as one of the top 10 candidates showing a strong negative association with drug resistance (Fig. 1d-e, Extended Data Fig. 1d). MAP2K6 expression was markedly higher in olaparib-sensitive cell lines and inversely correlated with IC50 values across the panel (Fig. 1f–g).

To validate these observations in clinical samples, we examined MAP2K6 expression in BRCA-wild-type (WT) ovarian tumors from patients who had received PARP inhibitor maintenance therapy between 2020 and 2023. Tumors from PARPi-sensitive patients (progression-free survival (PFS) for≥2 years) exhibited significantly higher MAP2K6 expression compared with those from resistant cases (PFS < 2 years), as determined by immunohistochemistry (Fig. 1h-i). Multivariate Cox regression, after univariable analysis, indicated that elevated abundance of MAP2K6 is an independent and potential predictive factor for improved prognosis in patients treated with PARP inhibitor (Extended Data Fig. 1e–f). Collectively, these findings identify MAP2K6 as a candidate biomarker predictive of olaparib sensitivity in BRCA-proficient ovarian cancer.

### MAP2K6 promotes PARP inhibitor sensitivity through PARP1-dependent mechanisms

**Fig. 2:**
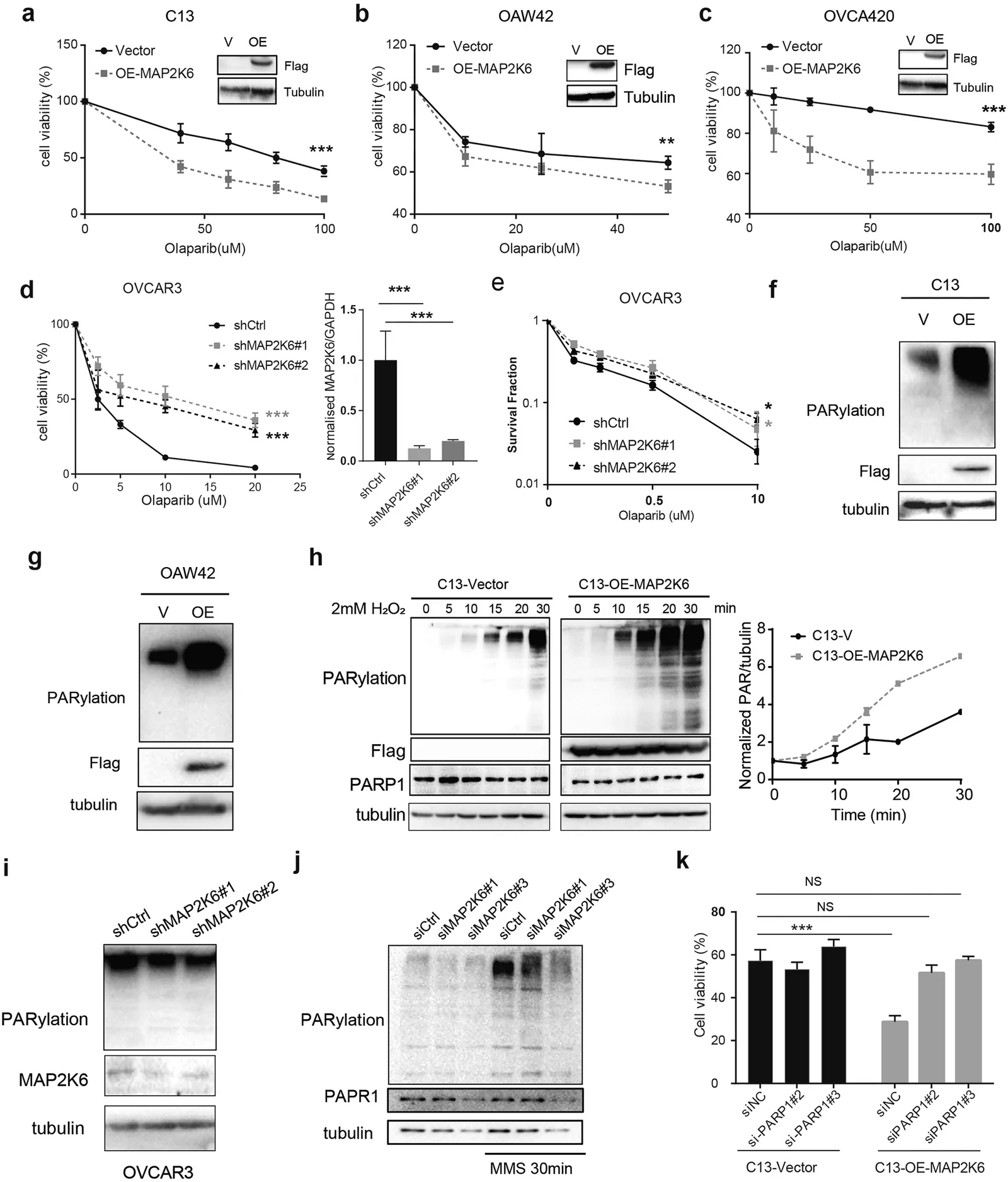
MAP2K6 modulates PARylation and olaparib response in ovarian cancer cells. **a**–**c** CCK8 cell viability of C13(a), OAW42(b), OVCA420(c) cells stably expressing Flag or Flag-MAP2K6. The cells were exposed to olaparib at indicated concentrations for 48 h and then analyzed by CCK8 assay (two-tailed unpaired Student’s *t* tests. **p < 0.01, ***p < 0.001, error bar = ±SEM, *n* = 6). Flag-MAP2K6 expression levels are shown. **d** CCK8 cell viability of OVCAR3 cells stably expressing shCtrl or shMAP2K6 following olaparib treatment for 28 h (two-tailed unpaired Student’s *t* tests. ***p < 0.001; *n* = 6; error bar = mean ± SEM). MAP2K6 mRNA levels are shown on the right. **e** Colony formation assays of OVCAR3 cells expressing shCtrl or shMAP2K6 treated with olaparib at the indicated doses (two-tailed unpaired Student’s *t* tests. *p < 0.05; *n* = 3; error bar = mean ± SEM). Western blots detecting PARylation in C13 (**f**) and OAW42 (**g**) cells stably expressing Flag (V) or Flag-MAP2K6 (OE). **h** Western blots of PARylation in C13 Flag(Vector) or Flag-MAP2K6(OE-MAP2K6) cells treated with 2 mM H_2_O_2_ for the indicated times. Quantification of PARylation normalized to tubulin from *n* = 3 independent experiments is shown on the right (error bar = mean ± SEM). **i** Western blots of PARylation and MAP2K6 in OVCAR3 cells expressing shCtrl or shMAP2K6. **j** Western blot analysis of PARylation in A2780 cells transfected with control or MAP2K6 siRNA, with or without 10 mM MMS treatment for 10 min, as indicated. **k** CCK8 viability of C13 cells stably expressing Flag(Vector) or Flag-MAP2K6(OE-MAP2K6) following MAP2K6 knockdown by siRNA, with or without olaparib (50 μM) treatment for 28 h (two-tailed unpaired Student’s *t* tests. ***p < 0.001; *n* = 6; error bar= mean ± SEM).

To determine the role of MAP2K6 in regulating the response to PARP inhibition, we performed both gain- and loss-of-function experiments in ovarian cancer cell lines exhibiting differential MAP2K6 expression. Stable overexpression of MAP2K6 in low-expressing C13, OAW42 and OVCA420 cells markedly enhanced olaparib sensitivity, resulting in reduced cell viability (Fig. 2a-c). Conversely, transient knockdown or stable depletion of MAP2K6 significantly increased cell viability and clonogenic survival following olaparib treatment (Fig. 2d, e and Extended Data Fig. 2a-d), indicating that MAP2K6 is required for the full cytotoxic response to PARP inhibition. These results establish MAP2K6 as a positive regulator of PARPi sensitivity in ovarian cancer cells.

To explore the mechanism by which MAP2K6 modulates PARPi response, we next assessed PARP enzymatic activity by measuring cellular PARylation levels, a direct readout of PARP1 activation. MAP2K6-overexpressing C13 and OAW42 cells displayed elevated PARylation both under basal conditions and following DNA damage induced by hydrogen peroxide (H_2_O_2_) treatment (Fig. 2f-h and Extended Data Fig. 2e). In contrast, MAP2K6 depletion markedly reduced PARylation both under basal conditions and following DNA damage induced by methyl methanesulfonate (MMS) or hydrogen peroxide (H_2_O_2_) treatment (Fig. 2i-j and Extended Data Fig. 2f), suggesting that MAP2K6 enhances PARP1 activity in ovarian cancer cells. Since MAP2K6 signaling is known to function through the p38 kinase cascade [20–22], we evaluated PARylation in the presence of pharmacological p38 inhibition. Treatment with the p38 inhibitors Adezmapimod or SD-202190 attenuated MAP2K6 overexpression-induced PARylation (Extended Data Fig. 2g-h), suggesting that MAP2K6 regulates PARP1 activity through the p38 pathway.

To determine whether MAP2K6-mediated sensitization to PARPi depends on PARP1, we silenced PARP1 in control and MAP2K6-overexpressing cells and evaluated their response to olaparib. PARP1 depletion had minimal effect on drug sensitivity in control cells but largely abolished the enhanced olaparib response conferred by MAP2K6 overexpression (Fig. 2k and Extended Data Fig. 2i-j). Similarly, depletion of p38 abrogated MAP2K6-induced sensitization to olaparib (Extended Data Fig. 2k), indicating that MAP2K6 promotes PARPi sensitivity through a PARP1- and p38-dependent mechanism.

To directly test whether MAP2K6 promotes PARP1 trapping, we performed chromatin fractionation assays to assess chromatin-bound PARP1 in cells with or without MAP2K6 overexpression under olaparib treatment. MAP2K6 overexpression increased the basal chromatin association of PARP1 compared with vector control cells. Upon olaparib treatment, MAP2K6-overexpressing cells exhibited a marked increase in chromatin-bound PARP1, consistent with enhanced PARP1 trapping. Notably, pharmacological inhibition of p38 significantly attenuated MAP2K6-induced chromatin association of PARP1 and reduced olaparib-induced PARP1 trapping (Extended Data Fig. 2l). Consistent with these obervations, transcription factors response elements (TFRE)-based proteomic profiling [24] across ovarian cancer cell lines revealed a positive correlation between MAP2K6 protein expression and the ability of PARP1 to associate with transcriptional DNA elements (Extended Data Fig. 2m-o), suggesting that MAP2K6 expression is also linked to the transcriptional regulatory activity of PARP1. Together, these findings demonstrate that MAP2K6 promotes PARP inhibitor sensitivity in a PARP1-dependent manner, likely through upregulation of PARP1 activity and PARylation in ovarian cancer cells.

### MAP2K6 promotes PARP1 activation through NAMPT-dependent NAD^+^ metabolism

**Fig. 3:**
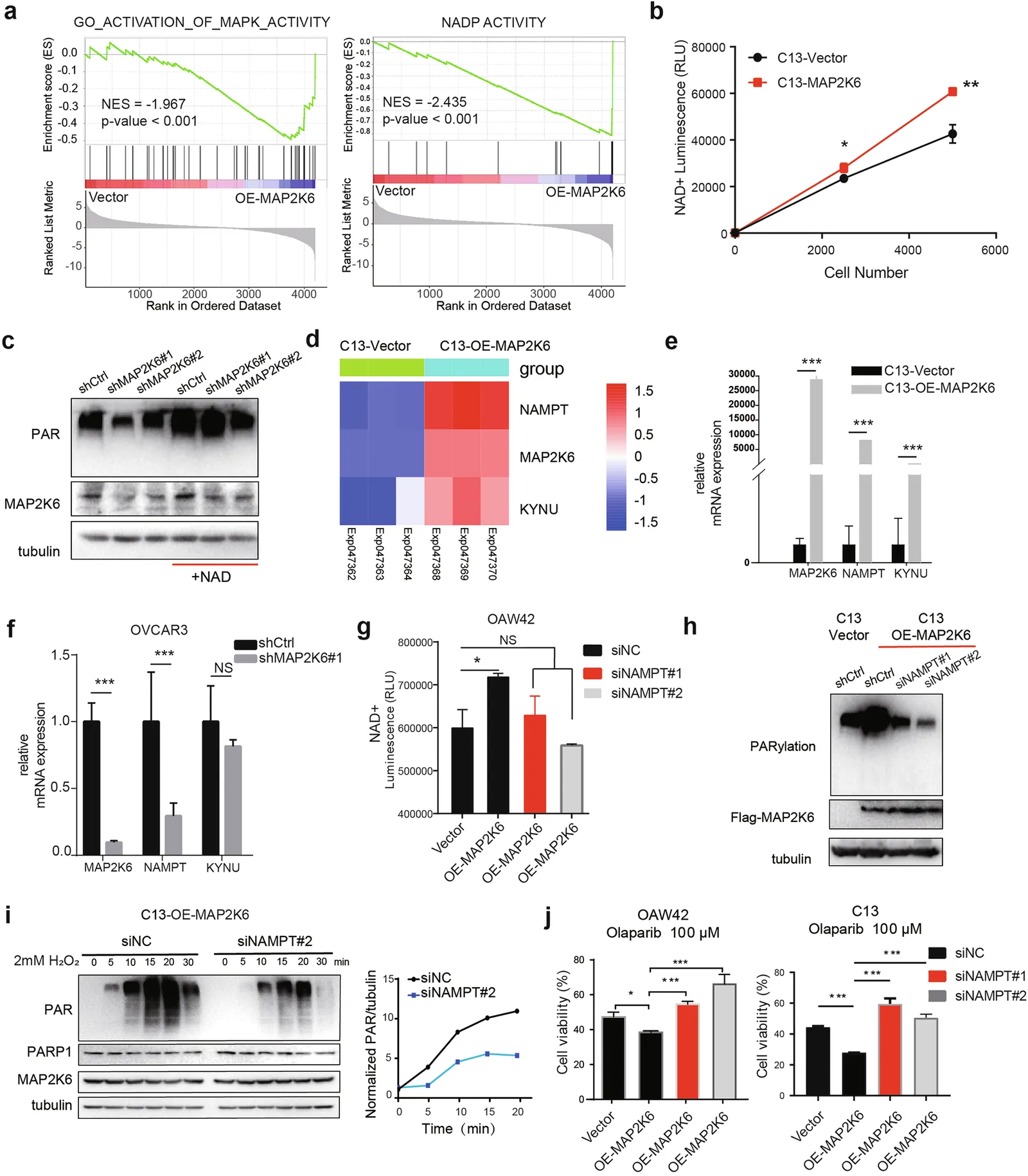
MAP2K6 promotes NAD^+^ synthesis and PARylation through the NAMPT pathway. **a** GSEA analysis showing enrichment of MAPK activity and NAD^+^ metabolic pathways in C13 Flag-MAP2K6-overexpressing cells compared with Flag cells. NES and P value are indicated. **b** Cellular NAD^+^ levels measured by the NAD/NADH-Glo™ assay in C13 Flag or Flag-MAP2K6 cells (two-tailed unpaired Student’s *t* tests. *p < 0.05, **p < 0.01, error bar = ±SEM, *n* = 3). **c** Western blot of PARylation level in OVCAR3 cell lines stably expressing shCtrl or shMAP2K6 treated with 100 μM NAD^+^ for 24 h. **d** Heatmap showing proteomic abundance of the indicated proteins in C13 Flag-MAP2K6 versus control (Vector) cells. Relative mRNA expression of MAP2K6, NAMPT, and KYNU measured by qPCR in C13 Flag-MAP2K6 (OE-MAP2K6) versus control (Vector) cells (**e**) and in OVCAR3 shMAP2K6 versus shCtrl cells (**f**). (two-tailed unpaired Student’s *t* tests. ***p < 0.001; error bar = mean ± SEM, *n* = 3). **g** Cellular NAD^+^ levels measured with NAD/NADH-Glo™ assay in OAW42 cells expressing Flag or Flag-MAP2K6 with NAMPT knockdown (two-tailed unpaired Student’s *t* tests. *p < 0.05, error bar = ±SEM, *n* = 3). **h** Western blot analysis of PARylation in C13 cell lines stably expressing Flag or Flag-MAP2K6 following NAMPT knockdown. **i** Western blots analysis of PARylation in C13 Flag-MAP2K6 cells with NAMPT knockdown treated with 2 mM H_2_O_2_ for the indicated times. Quantification of PARylation normalized to tubulin ratios is shown on the right. **j** CCK8 cell viability of OAW42 and C13 cell lines stably expressing Flag or Flag-MAP2K6 with NAMPT knockdown and treated with olaparib (100 μM) for 48 h (two-tailed unpaired Student’s *t* tests. *p < 0.05, ***p < 0.001, error bar = ±SEM, *n* = 6).

To determine how MAP2K6 regulates PARP1 activity, we performed proteomic profiling of C13 cells with or without MAP2K6 overexpression. Gene set enrichment analysis (GSEA) revealed significant enrichment of MAPK signaling and NAD^+^ metabolic pathways in MAP2K6-overexpressing cells (Fig. 3a). Consistent with these findings, MAP2K6 overexpression markedly increased the cellular NAD^+^ level, indicating enhanced NAD^+^ biosynthesis (Fig. 3b and Extended Data Fig. 3a-b). Inhibition of p38 significantly attenuated the MAP2K6-induced increase in NAD^+^ levels (Extended Data Fig.3c). Given that NAD^+^ serves as a substrate for PARP1-mediated PARylation, depletion of MAP2K6 in OVCAR3 cells led to a substantial reduction in global PARylation, which was restored upon supplementation with exogenous NAD^+^ (Fig. 3c). These data suggest that MAP2K6-p38 axis modulates PARP1 activation through NAD^+^ metabolism.

To identify the metabolic determinants underlying this NAD^+^ increase, we analyzed the expression of key enzymes in the NAD^+^ biosynthetic pathway. Proteomic profiling revealed that the rate-limiting enzyme nicotinamide phosphoribosyltransferase (NAMPT) and kynureninase (KYNU) were strongly upregulated in MAP2K6-overexpressing cells (Fig. 3d). Quantitative RT-PCR confirmed that MAP2K6 overexpression elevated NAMPT and KYNU transcript levels, whereas MAP2K6 knockdown suppressed their expression, with NAMPT showing the most pronounced change (Fig. 3e, f). Consistently, pharmacological inhibition of p38 diminished the MAP2K6-induced elevation of NAMPT mRNA levels, indicating that NAMPT upregulation occurs downstream of MAP2K6-p38 signaling (Extended Data Fig. 3d-e). Furthermore, NAMPT depletion abolished the MAP2K6-induced increase in NAD^+^ levels (Fig. 3g), indicating that MAP2K6 enhances NAD^+^ biosynthesis primarily through NAMPT upregulation.

Functionally, MAP2K6 overexpression promoted robust PARylation under both basal and DNA damage conditions (Fig. 2f-h and Extended Data Fig. 2e). This effect was completely abrogated by NAMPT knockdown (Fig. 3h-i and Extended Data Fig. 3f-h), demonstrating that NAMPT-dependent NAD^+^ production is essential for MAP2K6-driven PARP1 activation. To further assess the functional consequence of MAP2K6-NAMPT-PARP1 coupling, we examined the cellular response to PARPi. MAP2K6-overexpressing cells displayed enhanced sensitivity to olaparib, whereas concurrent NAMPT depletion conferred resistance (Fig. 3j). Collectively, these results identify MAP2K6 as a positive regulator of PARP1 activity through NAMPT-mediated NAD^+^ biosynthesis, thereby sustaining PARP1-dependent PARylation and sensitizing ovarian cancer cells to PARP inhibition.

### MAP2K6 regulates NAMPT expression through HMGA1 phosphorylation

**Fig. 4:**
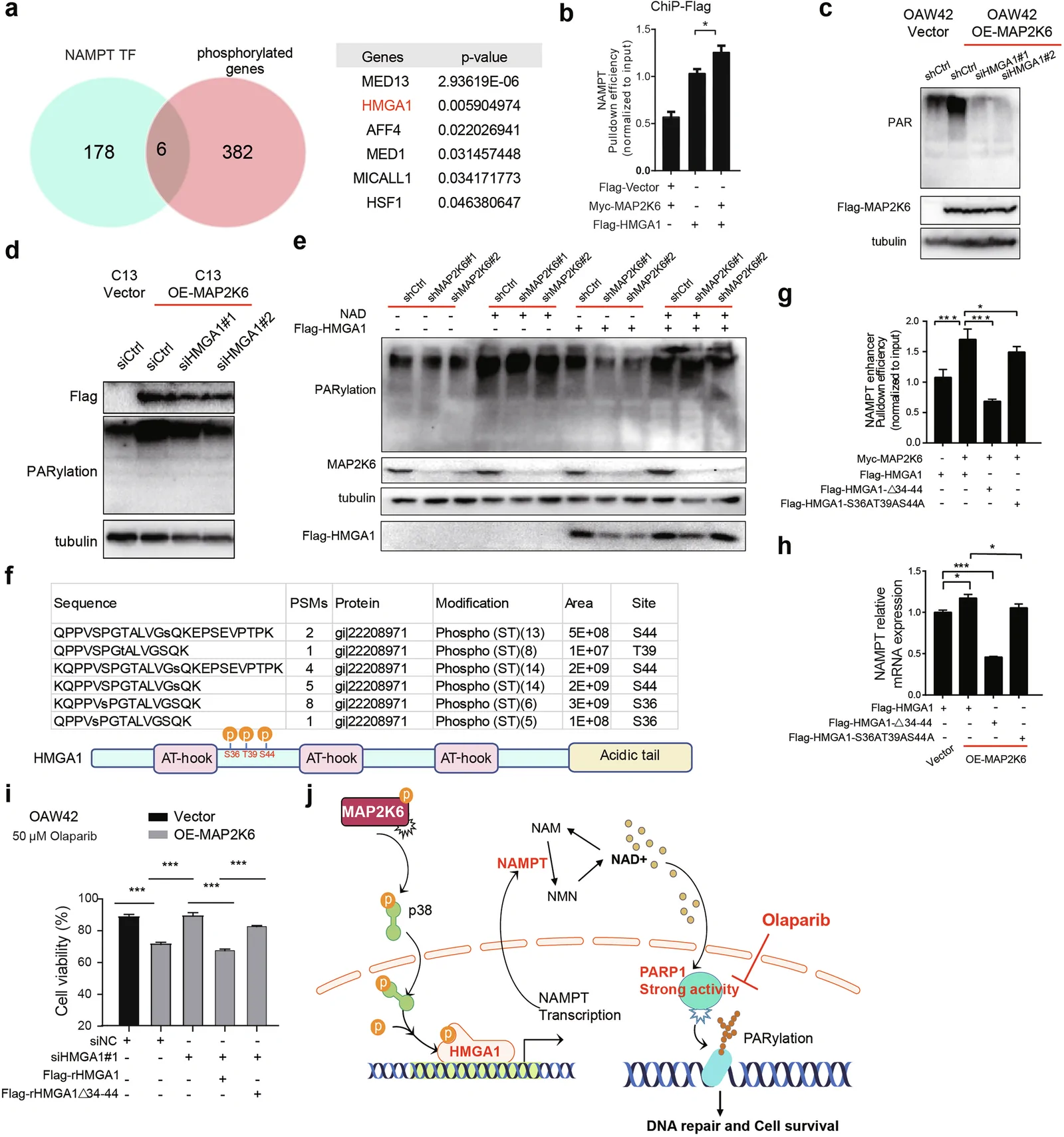
MAP2K6 signaling enhances NAMPT transcription via HMGA1. **a** Venn diagram showing the overlap of reported NAMPT transcriptional regulators with significantly altered phosphorylated proteins identified in C13 Flag-MAP2K6 versus control cells (*p* < 0.05, log_2_ (FC) > 0)). **b** Chromatin immunoprecipitation (ChIP) assays using anti-Flag antibodies in 293FT cells expressing the indicated plasmids. Pulled-down DNA was analyzed by qPCR using primers targeting the NAMPT enhancer. (two-tailed unpaired Student’s *t* tests. *p < 0.05; error bar = mean ± SEM, *n* = 3). Western blot analysis of PARylation in OAW42 (**c**) and C13 (**d**) cell lines stably expressing Flag or Flag-MAP2K6 with HMGA1 knocked down. **e** Western blots analysis of PARylation in OVCAR3 shCtrl or shMAP2K6 cells treated with 100 μM NAD^+^ for 24 h or transfected with Flag-HMGA1 for 24 h. **f** Candidate phosphorylation sites of HMGA1 identified in C13-MAP2K6-overexpressing cells. A schematic illustrating HMGA1 domain and phosphorylation site locations is shown below. **g** ChIP assays using anti-Flag antibodies in 293FT cells transiently transfected with Myc-MAP2K6 and indicated Flag-HMGA1 mutants. Pulled-down DNA was analyzed by qPCR targeting the NAMPT enhancer (two-tailed unpaired Student’s *t* tests. *p < 0.05, ***p < 0.001, error bar = ±SEM, *n* = 3). **h** Relative NAMPT mRNA expression in OAW42 cells stably expressing Flag or Flag-MAP2K6 and transiently transfected with the indicated HMGA1 mutants (two-tailed unpaired Student’s *t* tests. *p < 0.05, ***p < 0.001, error bar = ±SEM, *n* = 3). **i** CCK8 cell viability of OAW42 cells stably expressing Flag or Flag-MAP2K6 with HMGA1 knocked down or re-expression of siHMGA1#1-resistant HMGA1 mutants as indicated. Cells were treated with olaparib (50 μM) for 48 h (two-tailed unpaired Student’s *t* tests. ***p < 0.001, error bar = ±SEM, *n* = 6). **j** Schematic model illustrating the MAP2K6-p38-HMGA1-NAMPT axis and its role in enhancing PARylation and sensitizing ovarian cancer cells to olaparib. Statistical analyses were performed with the Student’s *t* test. *p < 0.05; **p < 0.01; ***p < 0.001; NS, Not Significant.

MAP2K6 is a dual-specificity kinase of the MAPK family that can activate transcriptional programs through phosphorylation of nuclear factors. To explore whether MAP2K6 regulates NAMPT expression by modulating transcription factor phosphorylation, we performed phosphoproteomic profiling in cells overexpressing MAP2K6. This analysis identified 388 significantly upregulated phosphorylated proteins (*t*.test, *p*-value <= 0.05; Fig. 4a and Extended Data Fig. 4a). Cross-referencing these phosphorylated proteins with known NAMPT transcriptional regulators revealed 6 candidates, among which HMGA1 ranked among the top three phosphorylated transcription factors (Fig. 4a). HMGA1 has previously been reported to control NAMPT transcription and promote NAD^+^ metabolism in senescent cells [25].

To validate these findings, we performed chromatin immunoprecipitation (ChIP) assays, which showed that MAP2K6 overexpression markedly enhanced HMGA1 occupancy at the NAMPT promoter (Fig. 4b). In contrast, pharmacological inhibition of p38 suppressed MAP2K6-induced HMGA1 promoter occupancy (Extended Data Fig. 4b), indicating that HMGA1 activation occurs downstream of MAP2K6-p38 signaling. Consistently, HMGA1 depletion abolished MAP2K6-induced global PARylation (Fig. 4c-d and Extended Data Fig. 4c-d). Ectopic expression of HMGA1 alone failed to rescue PARylation in MAP2K6-deficient cells, whereas supplementation with exogenous NAD+ restored PARylation levels (Fig. 4e), demonstrating that HMGA1 is required for MAP2K6-mediated PARP1 activation.

To determine which residues mediate MAP2K6-dependent HMGA1 activation, we examined phosphoproteomic data and detected phosphorylation of serine or tyrosine residues at positions S36, T39 and S44 within the link region of HMGA1 in MAP2K6-overexpressing cells (Fig. 4f). Phosphorylation-deficient HMGA1 mutants (S36A/T39A/S44A) slightly reduced MAP2K6-induced HMGA1 occupancy at the NAMPT promoter and NAMPT induction. Meanwhile, mutation of HMGA1 phosphorylation domain(Δ34–44 mutant) abolished the effect of MAP2K6 in promoting HMGA1 binding to the NAMPT enhancer and NAMPT transcription (Fig. 4g, h), suggesting that this region is essential for NAMPT transcriptional activation. Functionally, HMGA1 loss rendered MAP2K6-overexpressing cells resistant to the PARP inhibitor olaparib (Fig. 4i). Re-expression of siRNA-resistant wild-type HMGA1(rHMGA1), but not the Δ34–44 mutant, restored olaparib sensitivity (Fig. 4i, Extended Data Fig. 4e).

Together, these findings demonstrate that MAP2K6-p38 signaling activates HMGA1 by inducing phosphorylation-dependent structural changes in its linker region, thereby promoting NAMPT transcription, sustaining NAD^+^ biosynthesis, and enhancing PARP1-dependent PARylation (Fig. 4j).

### MAP2K6 enhances olaparib sensitivity and serves as a potential biomarker in ovarian cancer

**Fig. 5:**
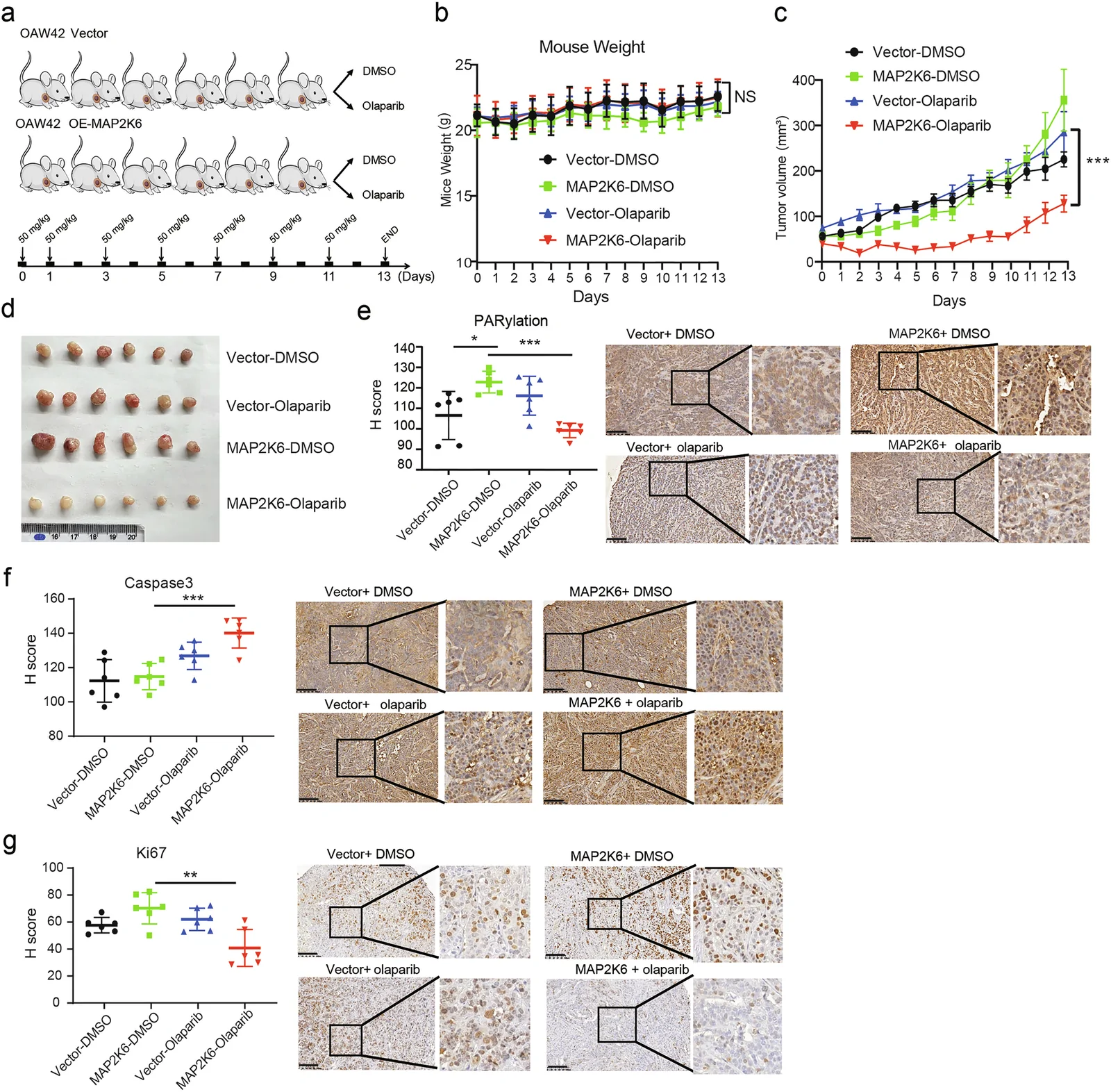
MAP2K6 enhances olaparib antitumor efficacy in vivo. **a** Schematic of the xenograft model and olaparib administration schedule used in nude mice. **b** Body weights of mice at the indicated time point. **c** Growth curves of xenograft tumors derived from OAW42 overexpressing Flag or Flag-MAP2K6 treated with DMSO or olaparib. The mice with tumors were randomly divided into four groups treated with DMSO or olaparib (50 mg/kg, i.p.) after the tumor volumes reached 60–80 mm^3^. Tumor volumes were measured at the indicated time points. **d** Tumors isolated from individual mice in each group. Representative immunohistochemical staining of PARylation (**e**), cleaved caspase-3 (**f**), and Ki-67 (**g**) in xenograft tumors (right). Scale bars, 100 μm. Quantification of H-scores from six mice in each treatment group is shown on the left. All the statistical analyses were performed with two-tailed unpaired Student’s *t* tests. *p < 0.05; **p < 0.01; ***p < 0.001, error bars= mean ± SEM, *n* = 6.

To determine whether MAP2K6 modulates the response to PARP inhibition in vivo, we established xenograft models using OAW42 ovarian cancer cells stably overexpressing MAP2K6 or control vector cells. Mice bearing these tumors were randomized into four cohorts with or without olaparib treatment (Fig. 5a). Body weights remained stable across all groups during the course of treatment (Fig. 5b). In the absence of olaparib, MAP2K6-overexpressing and control tumors displayed comparable growth kinetics. However, upon olaparib administration, MAP2K6-overexpressing tumors exhibited markedly enhanced therapeutic sensitivity, reflected by reduced tumor volume and delayed progression compared with controls (Fig. 5c,d). Tumor regrowth was observed following drug withdrawal, indicating that continuous olaparib exposure was required for sustained suppression.

To further evaluate the molecular effects of MAP2K6 on PARP inhibition response, we analyzed tumor sections for PARylation, Ki67, and cleaved caspase-3 expression. In untreated mice, MAP2K6-overexpressing tumors showed elevated PARylation relative to controls (Fig. 5e). Following olaparib treatment, both PARylation and Ki67 levels were significantly decreased, whereas cleaved caspase-3 was increased, indicating enhanced apoptosis and reduced proliferation in MAP2K6-overexpressing tumors (Fig. 5f, g).

Collectively, these results demonstrate that MAP2K6 promotes olaparib sensitivity in vivo by augmenting PARP-associated signaling and apoptotic response. These findings further suggest that MAP2K6 may serve as a candidate predictive biomarker to stratify ovarian cancer patients likely to benefit from PARP inhibitor therapy.

## Discussion

Tumor cells often exploit metabolic reprogramming to sustain their growth and survival under stress. Here, we identified MAP2K6 as a key signaling mediator that links metabolic rewiring to PARP activation in ovarian cancer. We found that MAP2K6 enhances PARP1 activity through transcriptional upregulation of NAMPT, the rate-limiting enzyme in the NAD^+^ salvage pathway, thereby expanding the intracellular NAD^+^ pool and promoting PARP1 activation and PARylation (Figs. 2 and 3). This signaling-metabolic coupling establishes MAP2K6 as a functional enhancer of PARP1 and a determinant of PARPi sensitivity.

Early studies reported that MAPK activation triggers PARP1 cleavage during apoptosis or that phosphorylated ERK can bind PARP1 to expose its NAD^+^-binding site [14, 19, 26]. Conversely, PARP1 has also been shown to function as an upstream regulator of MAPK signaling, modulating JNK and p38 MAPK pathways during oxidative stress-induced cell death [27]. These observations suggest the reciprocal crosstalk between PARP1 and MAPK signaling pathways. In contrast to these previously described mechanisms, our findings reveal that MAP2K6 signaling activates PARP1 by boosting NAD^+^ biosynthesis, providing a sustained substrate supply rather than transient modulation of its structure. This finding shifts the conventional view of PARP1 regulation from a transient, damage- or conformation-dependent response to a metabolically reinforced process driven by oncogenic signaling. Consistent with this, we observed that MAP2K6 expression is elevated in approximately 35% of ovarian cancers and positively correlates with NAMPT abundance in our proteomic cohorts (Extended Data Fig. 5a-b) [28] and publicly available datasets (Extended Data Fig. 5c) [29]. These observations suggest that the MAP2K6-NAMPT-PARP1 axis represents a broader, metabolism-linked mechanism of PARP1 activation with potential therapeutic relevance beyond HR-proficient malignancies.

Mechanistically, the transcriptional regulation of NAMPT by MAP2K6 appears to be mediated by HMGA1, a chromatin architectural protein whose DNA-binding conformation is dynamically modulated by phosphorylation. Our phosphoproteomic profiling identified three MAP2K6-dependent phosphorylation sites (S36, T39, and S42) within the linker regions connecting HMGA1’ s AT-hook motifs. Previous studies have shown that phosphorylation of these residues by IKBKE or PKC exerts divergent effects on HMGA1 localization and DNA-binding affinity [30, 31]. Our results reveal that mutations of these residues slightly reduced HMGA1 binding to the NAMPT promoter, whereas combined mutation of the entire linker region markedly disrupted DNA binding and NAMPT transcription. These findings suggest that the structural integrity of the HMGA1 linker region is critical for its DNA-binding conformation, thereby coupling MAPK signaling to metabolic gene regulation.

NAD+ homeostasis emerges as a central determinant of PARP1 activity and cellular stress tolerance. Since PARP1 consumes NAD^+^ to catalyze PARylation, fluctuations in NAD^+^ metabolism directly modulate its enzymatic activity. In most mammalian cells, NAD+ is primarily generated through the nicotinamide (NAM) salvage pathway, where NAMPT acts as the rate-limiting enzyme [32, 33]. Inhibition of NAMPT has been shown to deplete NAD^+^ and suppress PARP1-dependent PARylation [34, 35], whereas NAD^+^ supplementation can restore PARP1 activity and transcriptional function [36, 37]. Consistent with these observations, MAP2K6-driven NAMPT upregulation increased intracellular NAD+ levels and enhanced PARP1 activation, establishing a direct signaling–metabolic axis that links MAPK signaling to PARP regulation. This mechanism contrasts with canonical PARP activation, which is typically triggered by DNA lesions; instead, MAP2K6 signaling sustains PARP1 activity through metabolic reinforcement, providing a distinct layer of control over genome-maintenance pathways.

Collectively, our findings establish a model in which MAP2K6 reprograms NAD+ metabolism via the HMGA1–NAMPT axis to activate PARP1, thereby sensitizing ovarian cancer cells to PARP inhibition. This work expands the conceptual framework of PARP regulation from a purely DNA-damage-driven process to one governed by oncogenic signaling and metabolic state, providing new opportunities to extend PARP-targeted therapies. Future studies with larger patient cohorts and prospective clinical datasets will be required to validate MAP2K6 as a predictive biomarker for PARP inhibitor therapy. Further investigation of MAP2K6-dependent metabolic regulation may also reveal new strategies to target PARP activity and overcome resistance in ovarian cancer.

## Materials and methods

### Cell culture and reagents and clinical patient samples

Human ovarian cancer cell lines cultured in RPMI-1640 (Gibco, Catalogue No. 11875119) or DMEM (Gibco, Catalogue No. 11965092) medium supplemented with 10% fetal bovine serum (FBS) (Gibco, Cat# 10099141) at 37 °C with 5% CO₂. Stable MAP2K6-overexpressing or knockdown cell lines were generated using lentiviral transduction followed by puromycin selection (1 µg/mL). Olaparib (Selleck Chemicals, Cat# S1060), MMS (methyl methanesulfonate, Sigma, Cat# 129925-5 G), and H₂O₂ were dissolved according to the manufacturer’s instructions. The specimens used in Fig. 1h-i were collected from treatment-naïve ovarian cancer patients who underwent primary debulking surgery (*n* = 14) or diagnostic laparoscopy prior to neoadjuvant chemotherapy (NACT) (*n* = 5) between 2020 and 2023 at Xiangya Hospital of Central South University, China. All patients were confirmed to be BRCA wild-type. Following platinum-based chemotherapy, all patients received niraparib as maintenance therapy. Among these patients, three were classified as platinum-resistant, whereas sixteen were platinum-sensitive. Detailed clinical characteristics, including treatment history and platinum response status, have been shown in Table S1A. The study was approved by the Animal Ethics Committee of Xiangya Hospital Central South University (Changsha, China) and written informed consent was obtained from all participants.

### RNA interference, plasmid construction and shRNA mediated FBL knockdown

Small interfering RNAs (siRNAs) targeting MAP2K6(si1-sense(5’-3’): CCAAGGCUUGCAUUUCUAUTT; si2-sense(5’-3’): GCUCAUGGAUACAUCACUATT; si3-sense(5’-3’): CCUACAUACCCAGAGCUAATT), NAMPT(si1-sense(5’-3’): CCACCTTATCTTAGAGTTATT; si2-sense(5’-3’): GTAACTTAGATGGTCTGGAAT), HMGA1(si1-sense(5’-3’): ACUCCAGGAAGGAAACCAATT; si2-sense(5’-3’): ACUCCAGGAAGGAAACCAATT), and p38(si1-sense(5’-3’): GAGCUGAACAAGACAAUCUTT; si2-sense(5’-3’): GUCCAUCAUUCAUGCGAAATT; si3-sense(5’-3’): GGUCUCUGGAGGAAUUCAATT) as well as non-targeting controls (sense(5’-3’): UUCUCCGAACGUGUCACGUTT), were purchased from GenePharma. Transfections were performed using Lipofectamine RNAiMAX (Invitrogen, Cat# 13778150) following the manufacturer’s instructions. For MAP2K6 overexpression, human MAP2K6 cDNA was cloned into a pLVX-Flag-SBP-puro vector. For HMGA1 phosphorylation-deficient mutants, site-directed mutagenesis was performed using a ClonExpress II One Step Cloning Kit(vazyme, Cat# C112-01). The lentivirus technology was used for the knockdown or overexpression of MAP2K6. In brief, HEK293T cells were transfected by Viafect^TM^ with pMD2G, psPAX and pLKO-Tet-On containing shRNA targeting human MAP2K6 (shRNA#1: 5’- GAGCTAATGCAACATCCATTT-3’, shRNA#2: 5’- GGATCCGAGCCACAGTAAATA -3’) or shctrl shRNA. Viral supernatants were collected 48 hours after transfection. Cells were infected with lentivirus in the presence of polybrene (5 μg/ml, Sigma, Cat# TR-1003). The cells which had been stably transfected with shRNA targeting MAP2K6 or with shctrl shRNA were isolated by puromycin selection (1 μg/ml, Sigma, Cat# P9620) for 72 hours.

### RNA extraction and qPCR

Total RNA was isolated using TRIzol reagent (Invitrogen, Cat# 15596018CN). cDNA was synthesized with the PrimeScript RT reagent kit (Takara, Cat# RR037Q), and quantitative PCR (qPCR) was carried out using SYBR Green Master Mix (Bio-RAD, Cat# 1725124). Relative expression was normalized to GAPDH using the ΔΔCt method.

### Protein extraction, immunoblotting, and PARylation assay

Cells were lysed in lysis buffer (62.5 mM Tris PH 6.8, 2% SDS, 10% glycerol, 10 mM protease inhibitor cocktail (CWBIO, #CW2200S), 10 mM phosphatase A inhibitor (CWBIO, #CW2383), 1 mM PMSF (Sigma, #P7626)). SDS-PAGE loading buffer was added and heated to 100 °C for 10 minutes. For PARP1 trapping assay, cells were lysed in NETN buffer (20 mM Tris-HCl (pH 8.0), 100 mM NaCl, 1 mM EDTA, 0.5% NP-40, cocktail) and incubate on ice for 20 min. After centrifuge at 3000 g, 4 °C for 10 minutes; the supernatant were collected as the soluble fraction. Further clarify the supernatant by centrifugation at 12000 g, 4 °C for 10 min and wash the precipitate twice with NETN buffer, then add an appropriate amount of 0.2 M HCl and incubate on ice for 30 minutes. Ultrasonic disruption, centrifugation at 12,000×g for 10 minutes at 4 °C; collect the supernatant into a new EP tube, add 1/2 volume of 1 M pH 8.0 Tris-HCl to neutralize, and use this as the chromatin fraction. Protein concentrations were determined using a BCA assay (Thermo Fisher, 23225). Equal amounts of protein were separated by SDS–PAGE and transferred onto PVDF membranes. The membranes were incubated with primary antibodies against Flag (Sigma-Aldrich Cat# F1804, RRID:AB_262044), PARP1 (Santa Cruz Biotechnology Cat# sc-8007), PAR (Trevigen, Cat# 4335-MC-100), MAP2K6 (Proteintech, Cat# 12745-1-AP, AB_2141563), LaminA/C(Proteintech, Cat#. 81042-1-RR, AB_2923698), Histone3(H3, Abcam, Cat# ab1791) or p38(CST, Cat# 8690 s) overnight at 4°C. Then the membranes were washed with PBST 3 times and incubated with anti-mouse or anti-rabbit HRP-conjugated secondary antibodies (Cell Signaling Technology, CST) at room temperature for 1 h. Blots were visualized with the enhanced chemiluminescence (ECL) solutions (Millipore, #WBLLS0500) in ChemiDoc XRS machine (Bio-rad).

For immunoprecipitation (IP), HEK293T cells were transfected with indicated plasmids by lipo293, after 48 h, cells were harvested and lysed in lysis buffer A (50 mM Tris-HCl pH 7.5, 300 mM NaCl, 1 mM EDTA, 1%Triton, 10 mM protease inhibitor cocktail, 10 mM phosphatase A inhibitor, 1 mM PMSF, (for PARylation detection, 1 μM PARG inhibitor (TOCRIS, PDD0001723) were added in lysis buffer A)). After incubation on ice for 10 min with occasional mixing, then buffer B (50 mM Tris-HCl pH 7.5, 1 mM EDTA) was added with an equal volume of buffer A and incubated on ice for 20 min with occasional mixing. The lysates were centrifuged at 12 000 rpm at 4 °C for 10 min, collected supernatants and incubated with Streptavidin beads (GE healthcare, 17-5113-01) on a rotor overnight at 4 °C. The precipitated proteins were washed four times with IP washing buffer (20 mM Tris-HCl pH 7.0, 150 mM NaCl, 1 mM EDTA, and 0.2% triton) and then subjected to immunoblot analysis.

### Profiling, TFRE proteomics and phosphoproteomics LC-MS/MS analysis

Cells were washed for three times with prechilled PBS and then collected. Lysis was then carried out in 1% sodium deoxycholate, 10 mM Tris(2-carboxyethyl) phosphine, 40 mM 2-chloroacetamide and 100 mM Tris–HCl pH8.5 at 95 °C for 5 min and by 5 min sonication (3 s on and 3 s off, amplitude 25%). After 16,000 g centrifugation at 4 °C for 10 min, 100 μg proteins in the cleared lysate were digested overnight with 1:50 trypsin (Promega, USA). The next day, digestion was stopped by adding 1% formic acid. Precipitated sodium deoxycholate was removed by 10 min 16,000 g centrifugation at 4 °C and peptides in supernatant were desalted on C18 StageTips. Desalted peptides were vacuum-dried and stored at –80 °C until subsequent LC-MS/MS analysis.

TFRE-based proteomic analysis was performed as described previously [38]. Briefly, cells were suspended in 800 μl of Cytoplasmic Extraction Reagent I (CER I) buffer (NE-PER kit, Thermo Scientific). Nuclear proteins were extracted in accordance with the manufacturer’s instructions. Protein concentrations were determined using the Bradford method (Bio-Rad SmartSpec Plus, Bio-Rad Laboratories, Inc., USA). Approximately 2–3 pmol of biotinylated DNA was pre-immobilized on Dynabeads and then mixed with NEs from the tissues. The mixtures were supplemented with ethylenediaminetetraacetic acid/ethylene glycol-bis(β-aminoethyl ether)-N,N,N′,N′-tetraacetic acid to a final concentration of 1 mM, adjusted with NaCl to a total salt concentration of 200–250 mM, and then incubated for 2 h at 4 °C. The supernatant was discarded, and the Dynabeads were washed twice with NETN (100-mM NaCl, 20-mM Tris-Cl, 0.5-mM ethylenediaminetetraacetic acid and 0.5% [vol/vol] Nonidet P-40) and then twice with phosphate-buffered saline. The catTFRE pull-down beads were re-suspended with 20 μl of 1 × SDS loading buffer and boiled for 5 min. The samples were then loaded on 10-cm 10% SDS–polyacrylamide gel electrophoresis gels and run to 1/3 of the length. The gel was stained with Coomassie brilliant blue and then destained in 5% ethanol/10% acetic acid solution. Six bands were excised according to the molecular weight ranges and then subjected to in-gel trypsin digestion.

Phosphopeptide enrichment: a total of 0.5 mg cell protein lysates were digested with trypsin for phosphorylated peptides enrichment. 0.15 ml binding buffer (80% ACN, 5% trifluoroacetic acid (TFA (Sigma-Aldrich)), and 1 M lactic acid (Sigma-Aldrich)) was added to the digested peptides. Vortex vigorously at room temperature for 1 min and centrifuged 10 min at 14,000 g. Supernate was transferred to a new tube and 3 mg TiO_2_ beads were added. Incubation was processed at room temperature for 30 min on rotor with middle speed. Then beads were collected by centrifugation for 2 min at 2000 × g, and the supernatant was used to repeat the enrichment for twice with new TiO_2_. Beads were combined and wash in a clear tube with 500uL wash buffer (50% ACN and 5% TFA) for 5times. After that, beads were transferred into a 0.2 ml StageTip with two pieces of C18. Another twice wash was performed in tip with 100uL wash buffer per time. Phosphorylated peptides were eluted five times with 100uL elution buffer (40% ACN, and 18% aqua ammonia) per time. Peptide samples were concentrated in a SpeedVac.

For LC-MS/MS analysis, a Fusion mass spectrometer (Thermo Fisher Scientific) was coupled on-line to an Easy-nLC 1000 HPLC nanoflow system (Thermo Fisher Scientific). The vacuum-dried peptides redissolved in 0.1% formic acid were loaded onto an in-house packed reversed-phase C18 precolumn (2 cm × 100 μm; particle size, 3 μm; pore size, 120 Å) and then separated by a 150 μm × 30 cm silica microcolumn (homemade; particle size, 1.9 μm; pore size, 120 Å) with a linear gradient of 6–40% Mobile Phase B (acetonitrile and 0.1% formic acid) at a flow rate of 600 nl/min for 150 min. To acquire mass spectra, data-dependent mode was applied by carrying out a Full MS scan (AGC target 3 × 106 ions, maximum injection time 20 ms, 300–1400 m/z, R = 60,000 at 200 m/z) followed by up to 20 tandem MS/MS scans with high-energy collision dissociation (target 2 × 10^3 ^ions, max injection time 40 ms, isolation window 1.6 m/z, normalized collision energy of 27%), detected in the Ion trap (R = 15,000 at 200 m/z). Dynamic exclusion time was set to 18 s. All data was acquired using the Xcalibur software (Thermo Fisher Scientific).

### Chromatin immunoprecipitation (ChIP) assay

1×10^7^ HEK293 cells were transfected with indicated plasmids for 48 h and then crosslinked with 1% formaldehyde for 10 min. After reaction, the glycine was added at the final concentration of 0.125 M to quench the crosslink for 5 min. Cells were washed twice with cold PBS and harvested by scrapping in 1.5 ml PBS. Then, the cells were pelleted by centrifugation and resuspended in 2 ml lysis buffer I (10 mM HEPES at pH 6.5, 10 mM EDTA, 0.5 mM EGTA, 0.25% Triton X-100 supplemented with phosphatase/protease inhibitors) with consistent rotation at 4 °C for 10 min. After incubation, cells lysates were pelleted by centrifugation and then resuspended in 2 ml lysis buffer II (10 mM HEPES at pH 6.5, 1 mM EDTA, 0.5 mM EGTA, 200 mM NaCl and phosphatase/protease inhibitors). After centrifugation, nuclei pellets were added with 300 μl lysis buffer III (50 mM Tris at pH 8.1, 10 mM EDTA, 1% SDS and phosphatase/protease inhibitors) and sonicated to shear DNA to average sizes of 200-500 bp and then centrifuged. The supernatants were diluted 1:10 with buffer (10 mM Tris at pH 8.1, 150 mM NaCl, 2 mM EDTA and 1% Triton X-100) and incubated with 2 μg indicated Streptavidin beads (GE healthcare, 17-5113-01) at 4 °C with consistent rotation for overnight. Beads were washed 4 times with wash buffer (50 mM HEPES -KOH, pH 7.6, 500 mM LiCl, 1 mM EDTA, 1% NP-40, 0.7% Na-Deoxycholate). Following the washing, the immunoprecipitated DNA fragments were eluted with 1% SDS and 100 mM NaHCO3 for 15 min, and the crosslink was reversed by incubation at 65 °C for 3 h in the presence of RNaseA. The proteinase K were then added and the samples were incubated for another 2 h at 55 °C. The immunoprecipitated DNA and input DNA were purified with Cycle-Pure Kit (Omega, Cat#D6492-02*). Eluted DNAs were measured the concentrations and the same amount of DNA were analyzed by Real-time Q-PCR using primers located to two NAMPT enhancer region.

### NAD^+^ quantification

Intracellular NAD+ levels were measured using the NAD/NADH-Glo™ Assay Kit (Promega Cat #G9071) according to the manufacturer’s instructions. Cells were centrifuged and resuspended in PBS at a density of 4 × 10^5^ cells/ml. After twofold serial dilutions in PBS, 50 µl of diluted cells was transferred to each well of a white 96-well plate. Cells were lysed by adding 50 µl of bicarbonate base buffer with 1% DTAB. To measure NAD^+^, add 25 µl of 0.4 N HCl to 50 µl lysed cells and heat at 60°C for 15 minutes. Then incubate at room temperature for 10 minutes. Add 25 µl of Trizma^®^ base to each well of acid-treated samples and Measure NAD^+^ using the NAD/NADH-Glo™ Assay.

### Cell viability and colony formation assay

5000 Cells were seeded in 96-well plates and treated with indicated concentrations of olaparib (Selleck Chemicals, S1060). Cell viability was assessed after 48 h using the CCK8 assay (Bimake, #B34304). IC50 values were calculated by nonlinear regression using GraphPad Prism 9. Colony formation assay was performed with the indicated cell lines by plating 300 cells in 35 mm dishes. After 12 days, Cells were washed 3 times with PBS, fixed and stained with 0.3% crystal violet in methanol. The colonies were counted and normalized to untreated cells.

### Xenograft models

All animal studies were approved by the Institutional Animal Care and Use Committee of Central South University (XY20250207003). Female nude mice (6–8 weeks old, Hunan SJA Laboratory Animal Co., Ltd.) were subcutaneously injected with 2 × 10⁶ OAW42 cells stably expressing control vector or MAP2K6. When tumors reached 60 ~ 100 mm³, mice were randomized into groups receiving vehicle(10%DMSO / 30%PEG300/saline solution), olaparib (50 mg/kg, once every other day by intraperitoneal injection). Tumor volumes and body weight were measured everyday with calipers (volume = length × width² × 0.5). At study endpoint, tumors were excised, fixed, and processed for IHC. The investigator was fully blinded to the group allocation during the experiment.

### Immunohistochemistry (IHC)

Briefly, Paraffin-embedded specimens were cut into 4 μm sections and incubated at 65 °C for 1 h. Sections were deparaffinized with turpentine and rehydrated. Heat-mediated antigen retrieval was performed in Sodium Citrate buffer (3.0 g sodium citrate and 0.4 g citric acid in 1 L ddH2O, pH 6.0) for 30 minutes. Then, 3% endogenous peroxidase blocking solution and 3% normal goat serum blocking solution were used to block endogenous target activity and nonspecific sites. Slides were incubated with primary antibodies against MAP2K6 (Proteintech, Cat# 12745-1-AP, AB_2141563), Ki67 (MXB biotechnologies, #MAB-0672), Caspase-3(Proteintech, Cat#19677-1-AP), and PAR(Abcam, Cat#ab14459) overnight at 4 °C. Tissue sections were stained using Dako EnVision+ Dual Link system-HRP (DAB + ) kit. Slides were dehydrated with sequential alcohol (75%-80%-90%-95%-100%-100%), cover-slipped with neutral resin and observed under microscope(3 fields of view were selected randomly). Expression of the stained markers was scored using a histologic score (H score).

### Statistical analysis

MS raw files were processed with the Firmiana proteomics workstation [21]. Briefly, raw files were searched against the NCBI human Refseq protein database (released on 04–07–2013, 32,015 entries) in Mascot search engine (version 2.3, Matrix Science Inc.). The mass tolerances were 20 ppm for precursor and 50 mmu or 0.5 Da for product ions collected either by Fusion, respectively. The proteolytic cleavage sites are KR. Up to two missed cleavages were allowed. The database searching considered cysteine carbamidomethylation as a fixed modification and Acetyl (Protein N-term), oxidation of methionine as variable modifications. All identified peptides were quantified in Firmiana with peak areas derived from their MS1 intensity. Peptide FDR was adjusted to 1%. In the analysis of global proteomes, three or more high-confidence peptides (mascot ion score > 20) were required for protein identification, and also proteins with two high-confidence peptides (mascot ion score > 20) were included when any of these two peptides belongs to unique peptide. To achieve protein quantification, we used a label-free, intensity-based absolute quantification (iBAQ) approach [22]. The fraction of total (FOT), calculated by a protein’s iBAQ divided by total iBAQ of all identified proteins in one experiment, was used to represent the normalized abundance of a protein across experiments. For easy representation, the FOT was then multiplied by 105 to obtain iFOT [23]. Missing values were substituted with zeros.

Data are presented as mean ± SEM from at least three independent experiments. Statistical analyses were performed using GraphPad Prism 9. Comparisons between two groups were made using unpaired two-tailed Student’s *t* tests. *P*-values < 0.05 were considered statistically significant. Principal component analysis, correlation analysis and Kaplan-Meier survival analysis were performed in R program. Gene Set Enrichment Analysis was performed using the GSEA software.

## Supplementary information

supplementary figure legends

Figure S1

Figure S2-1

Figure S2-2

Figure S3

Figure S4

Figure S5

Table S1

origin western

## Data Availability

All other data generated or analyzed during this study are included in this published article and its supplementary information files. Requests for resources, raw data, or further information should be directed to and will be fulfilled by the corresponding author, R.T. (rongtan@csu.edu.cn). This study did not generate new, unique reagents.
