# Supplementary material for: MAP2K6 reprograms NAD+ metabolism via the HMGA1–NAMPT axis to sensitize ovarian cancer cells to PARP inhibition
Source: Cell Death Dis. 2026 Jul 17;17(1):880. doi: 10.1038/s41419-026-09104-2 (PMC13658224; doi:10.1038/s41419-026-09104-2)

Fig2a-c

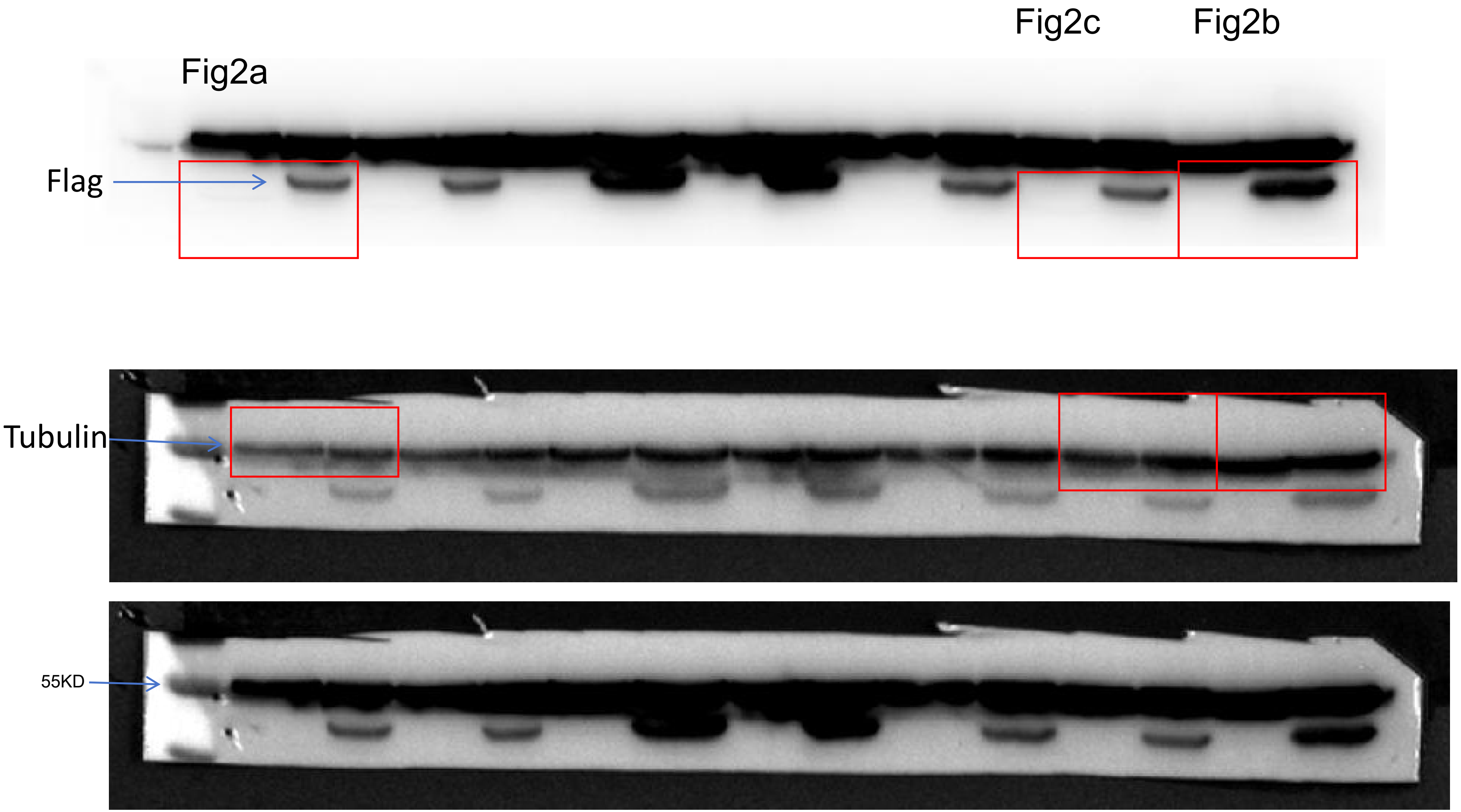

Fig2f

PAR

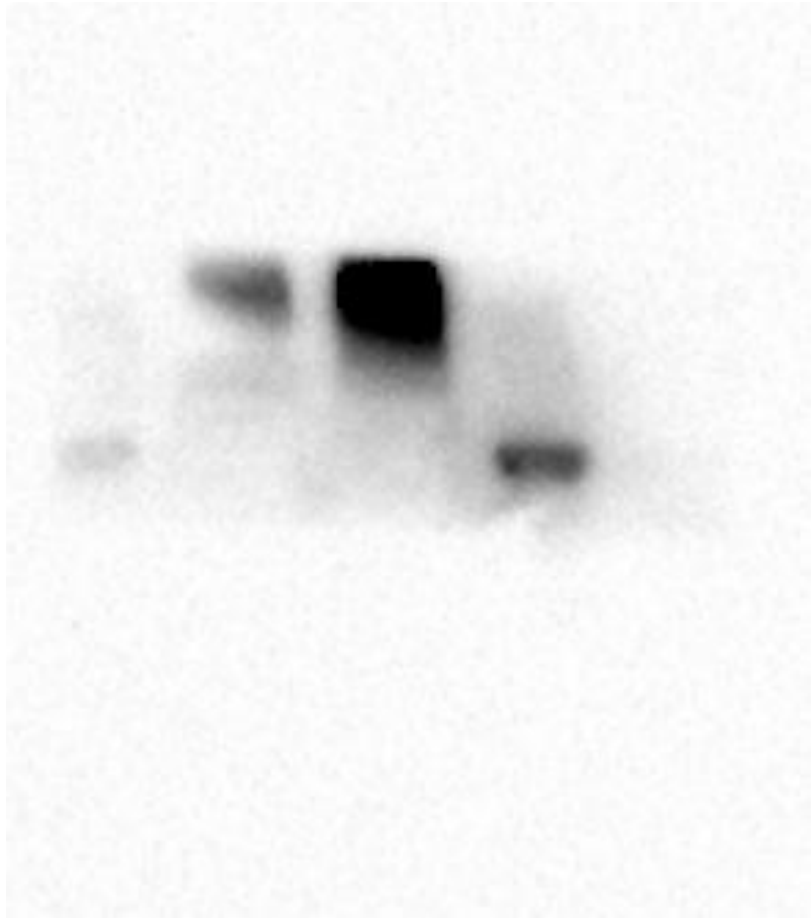

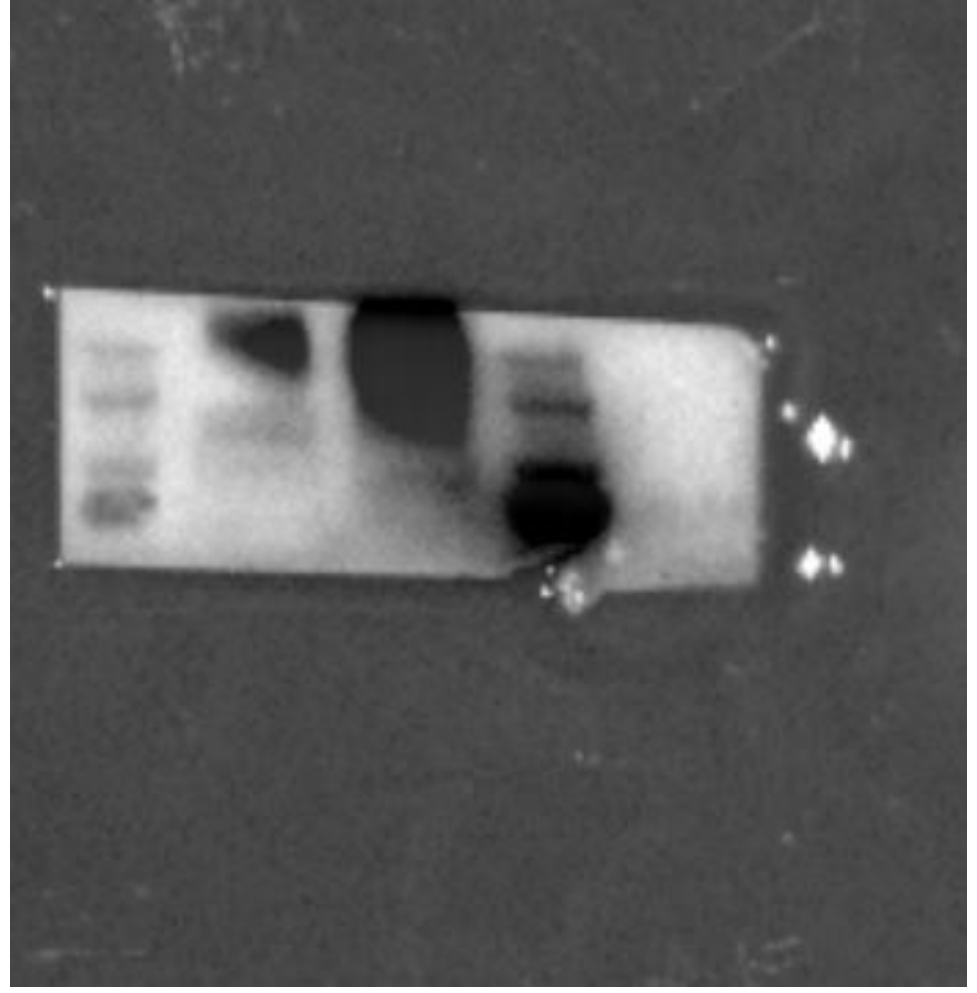

Flag

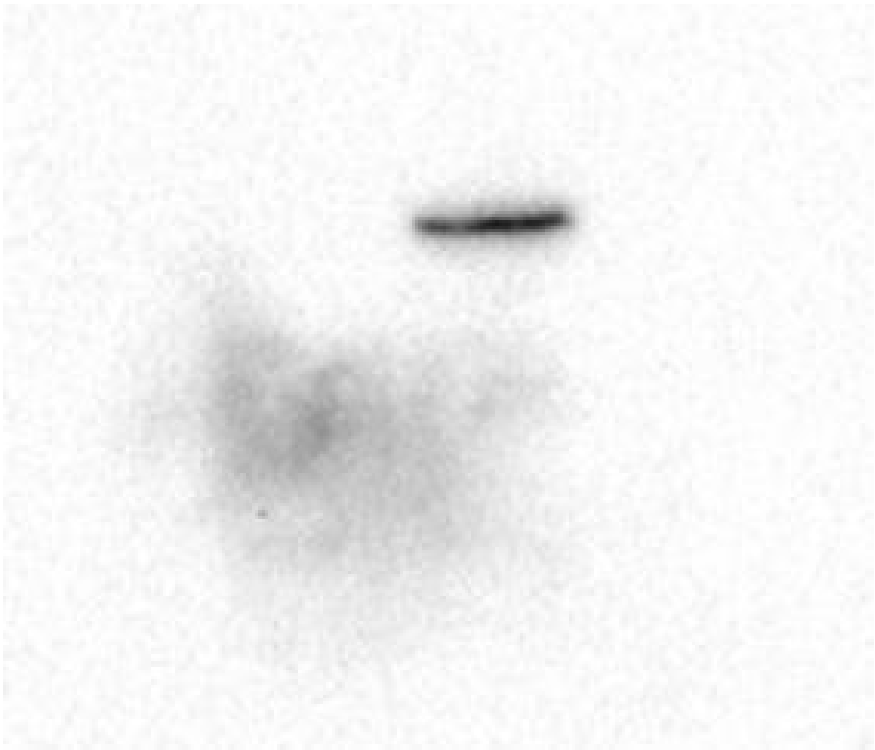

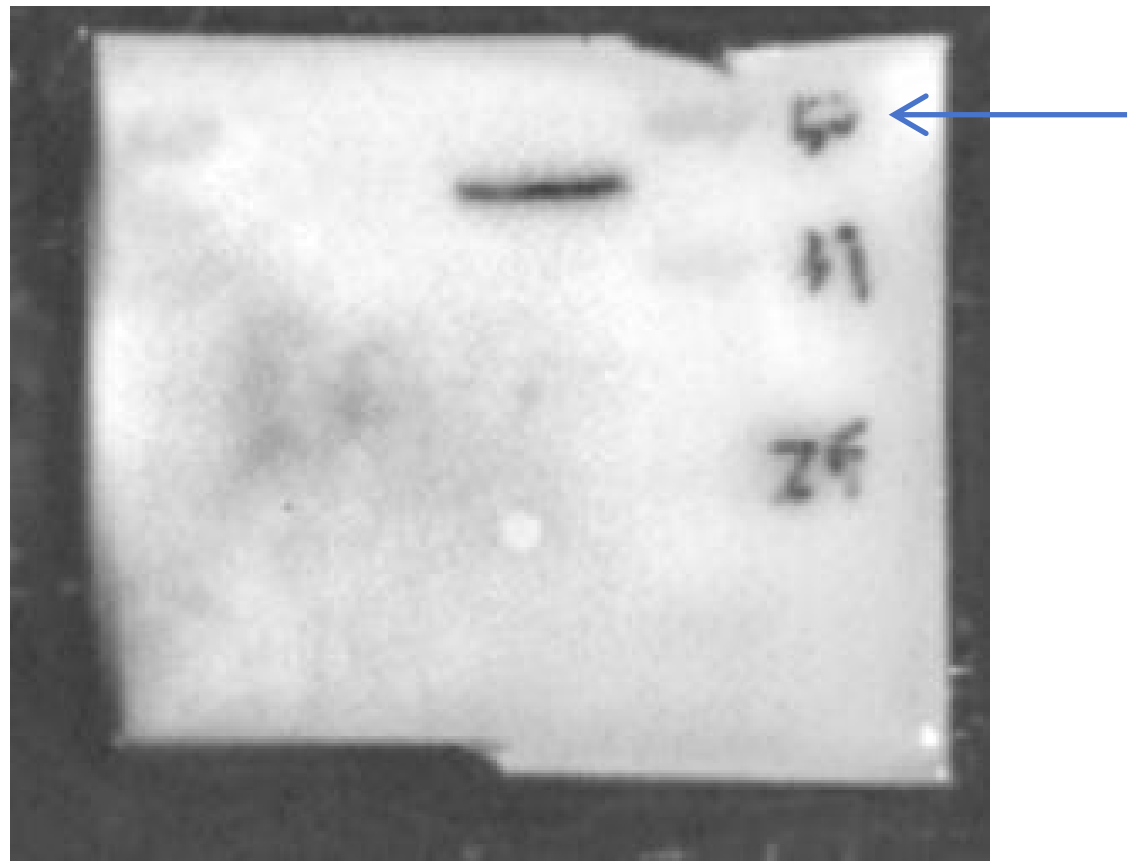

Tubulin

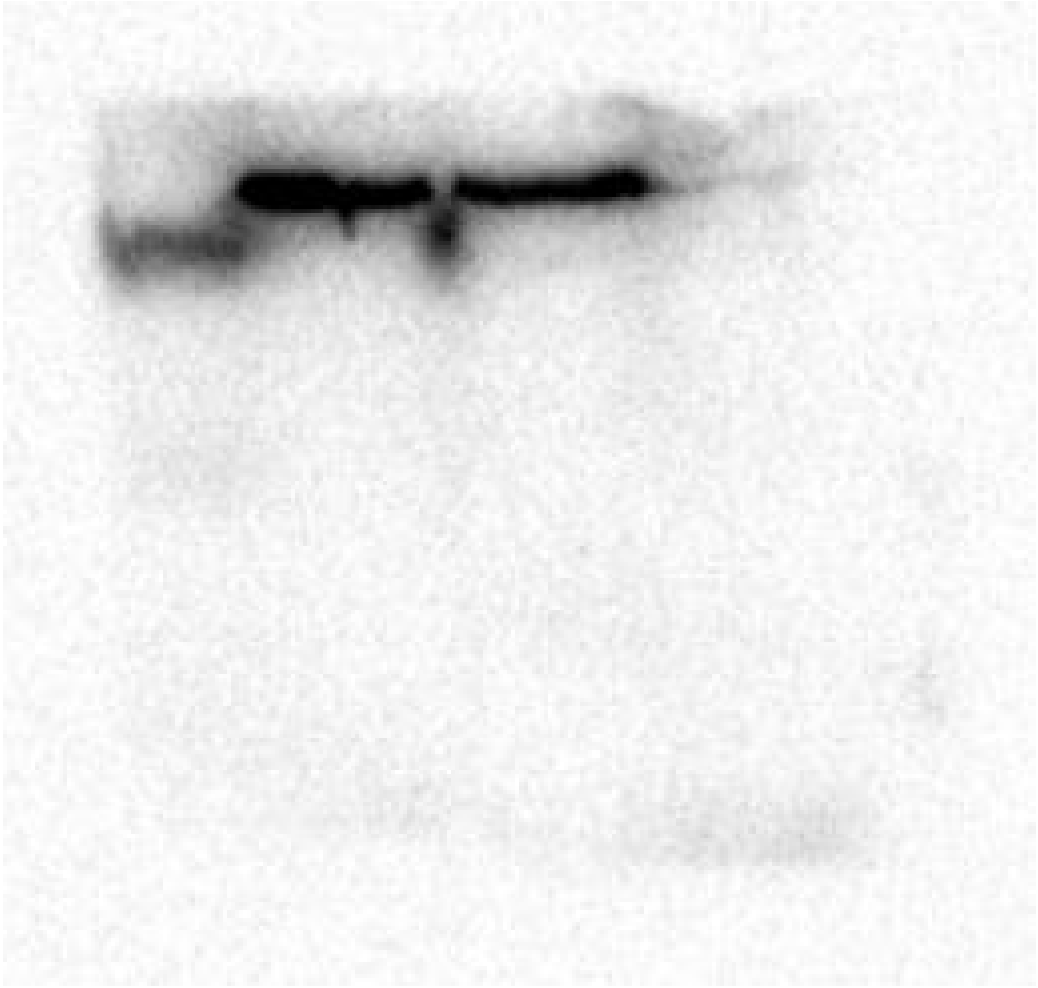

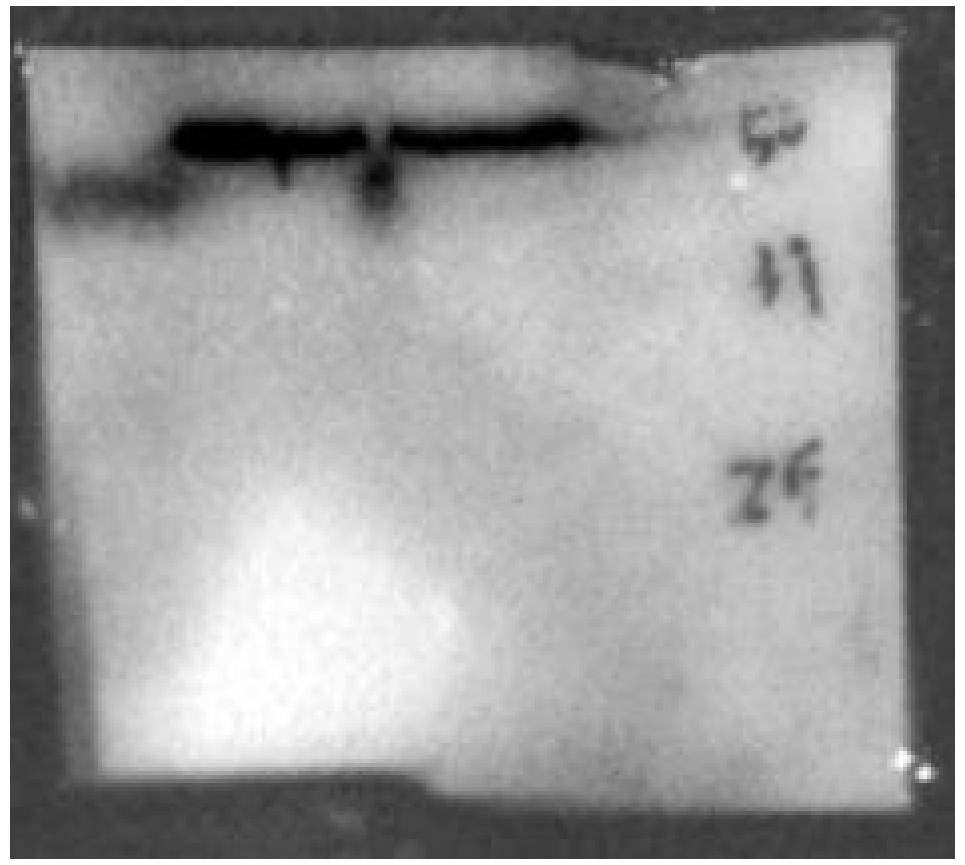

Fig2g

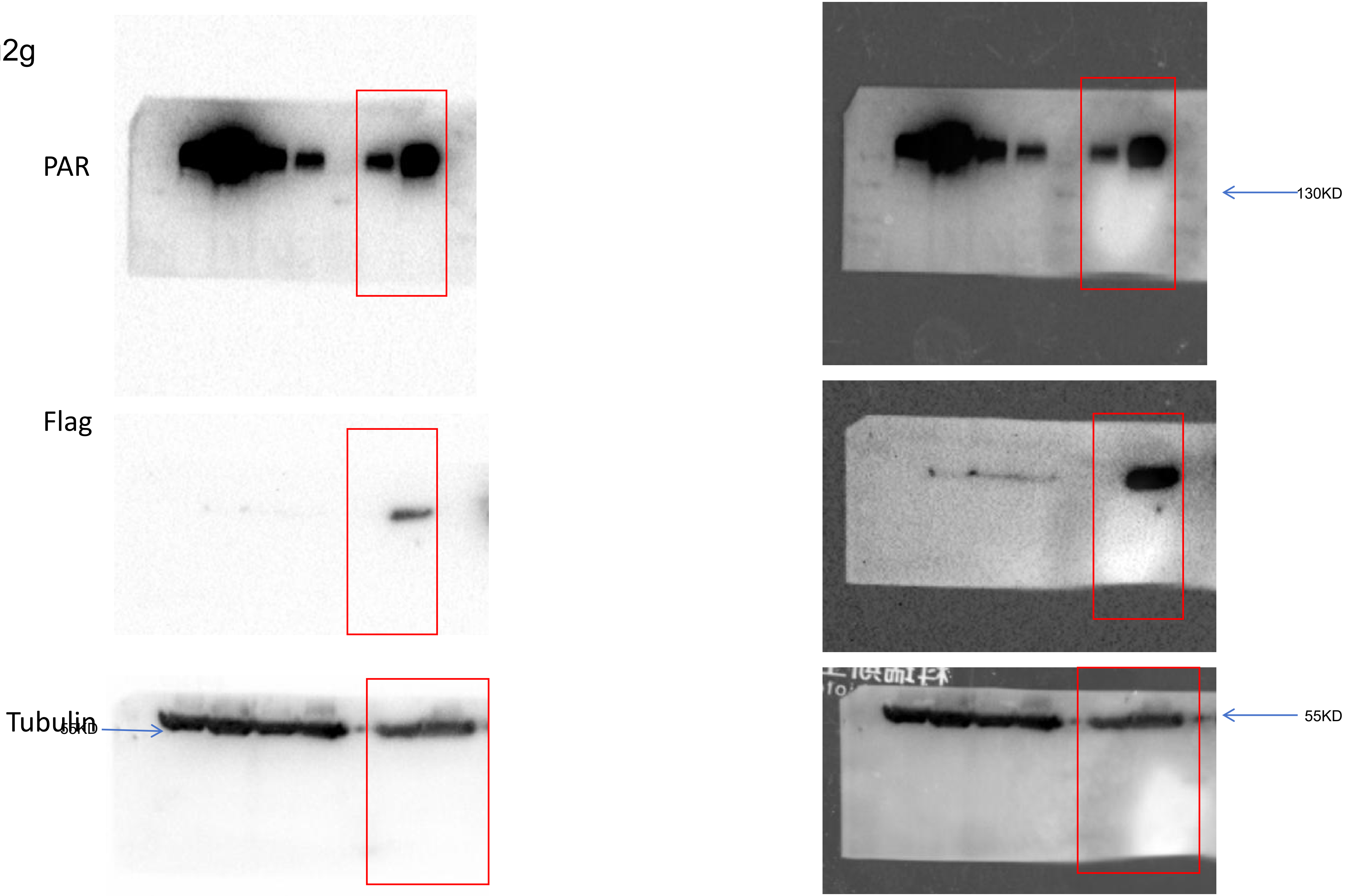

Fig2h

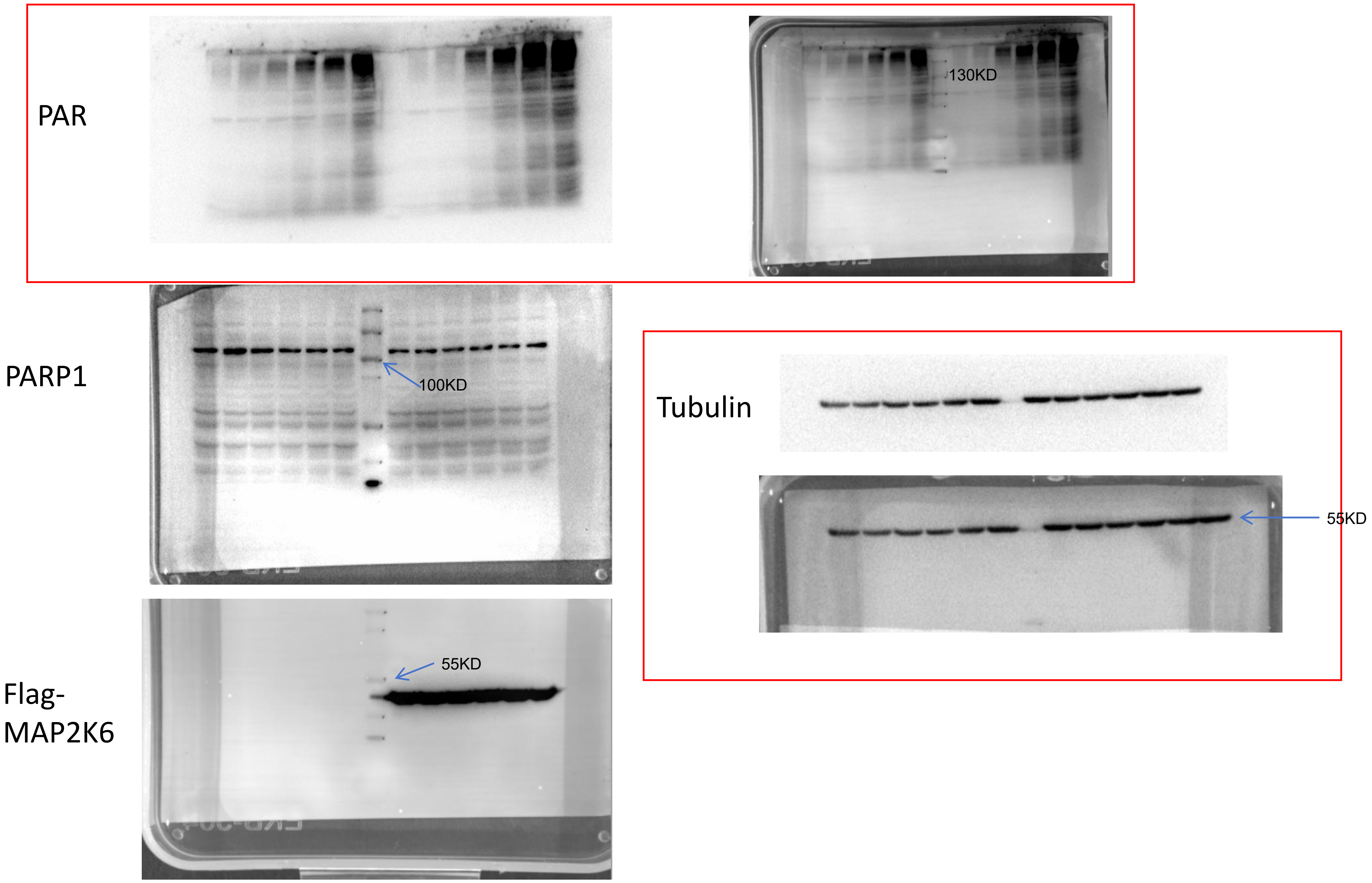

Fig2i

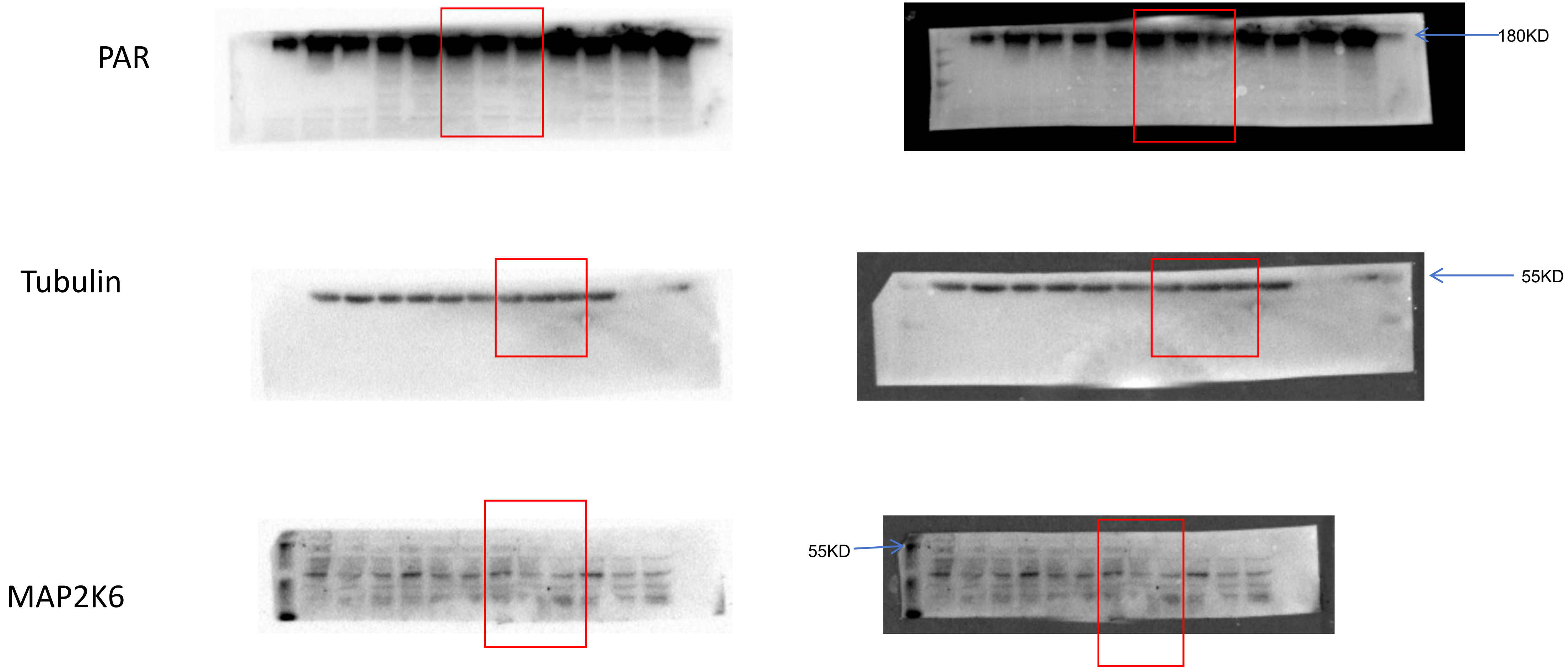

Fig2j

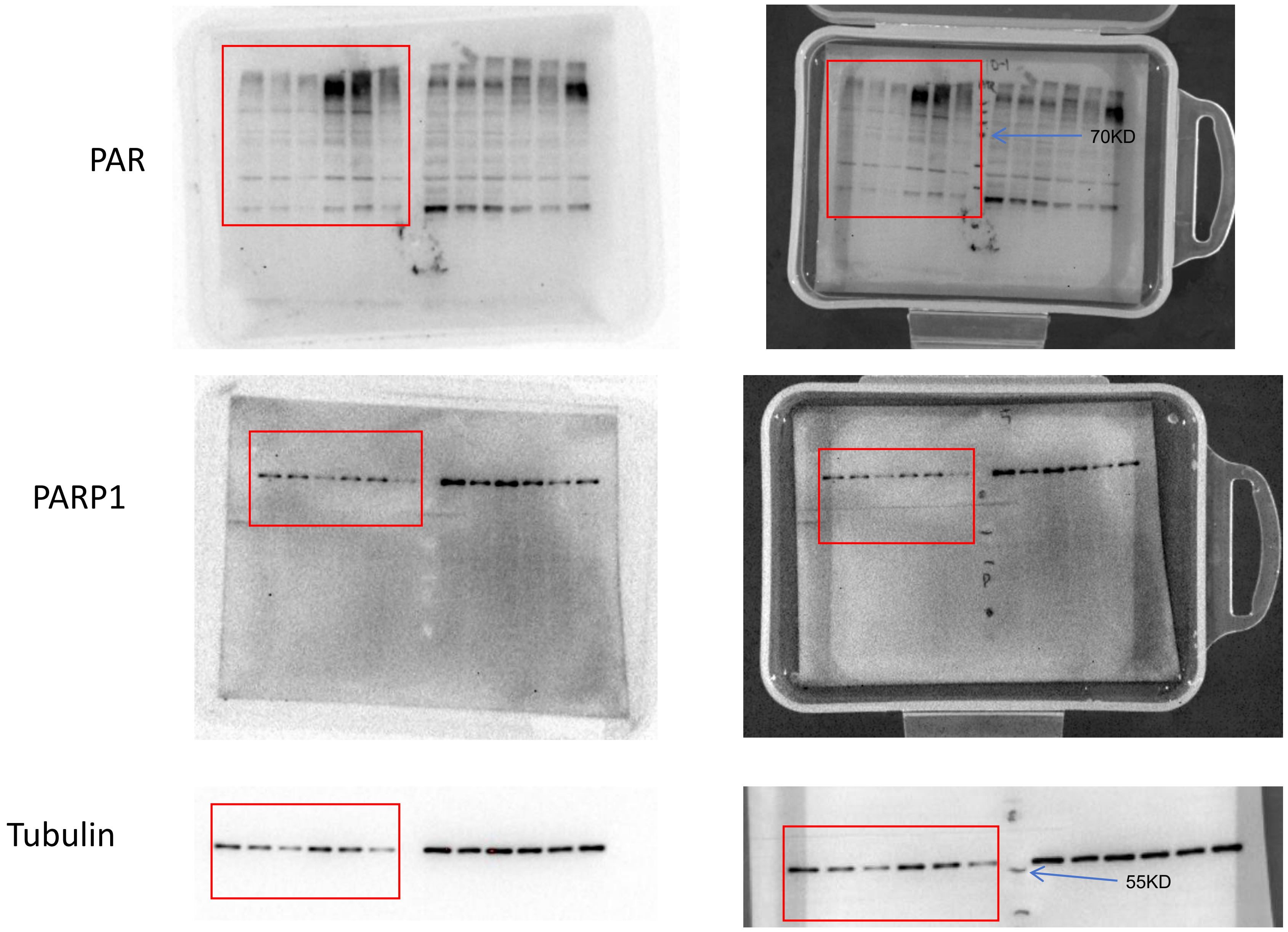

Fig3c

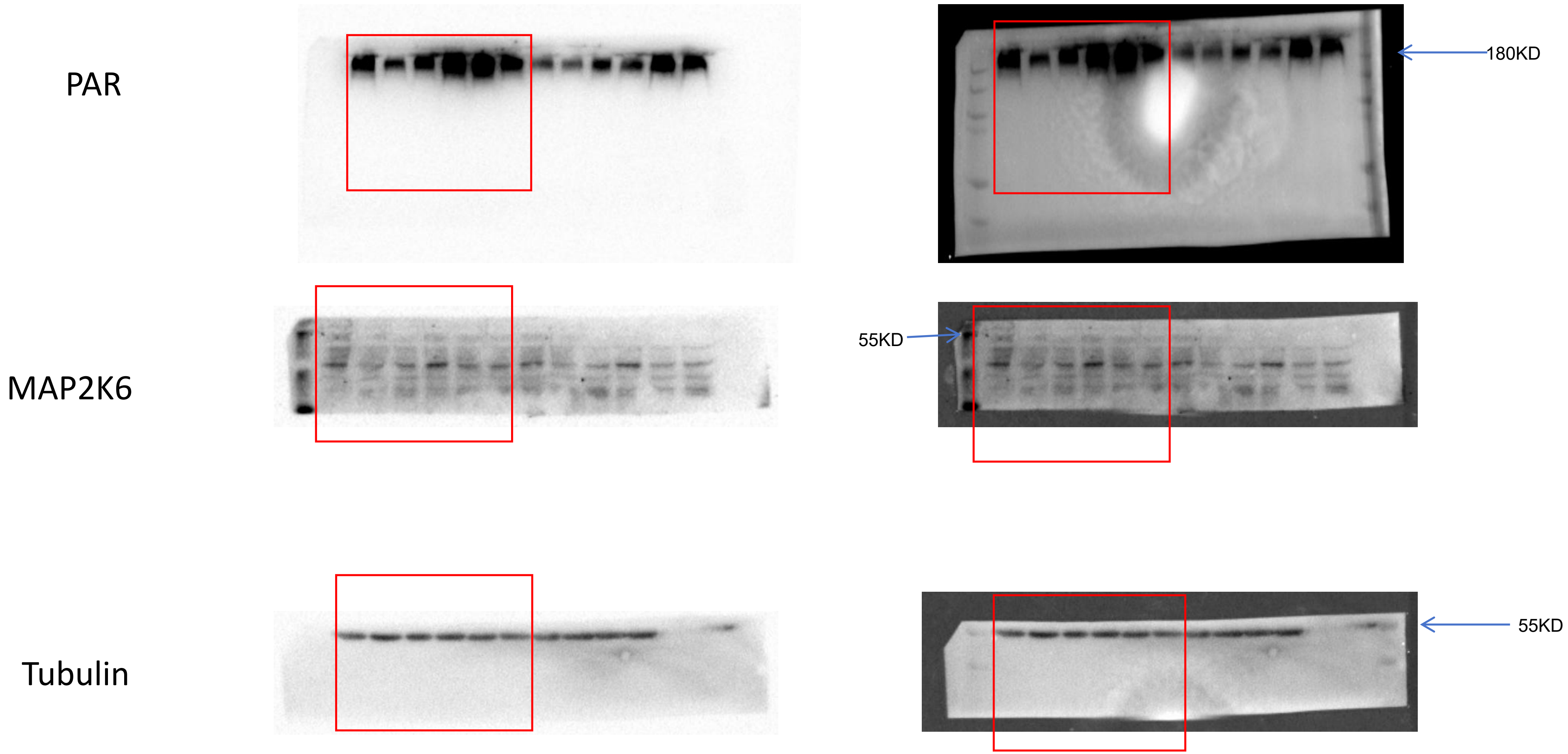

Fig3h

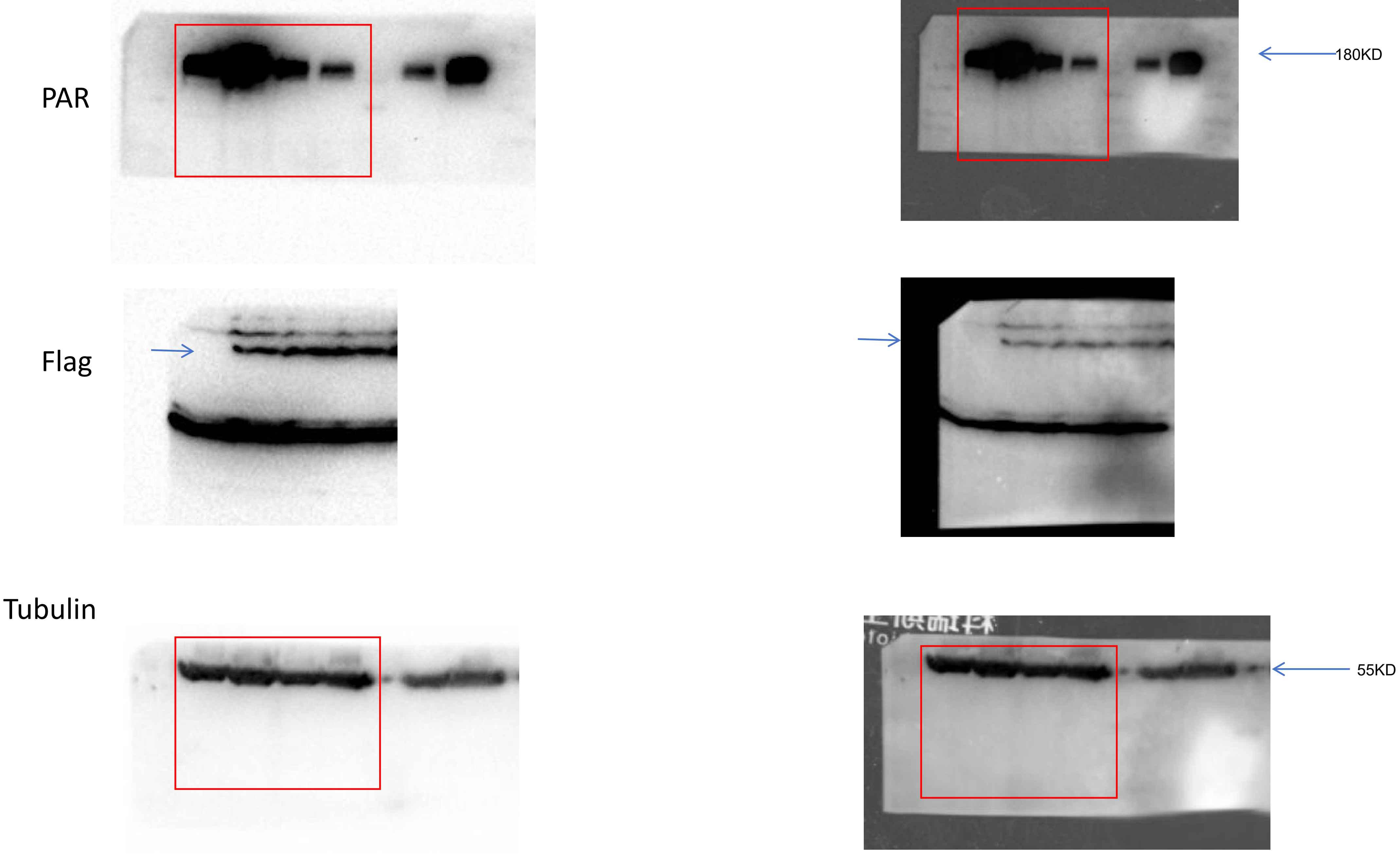

Fig3i

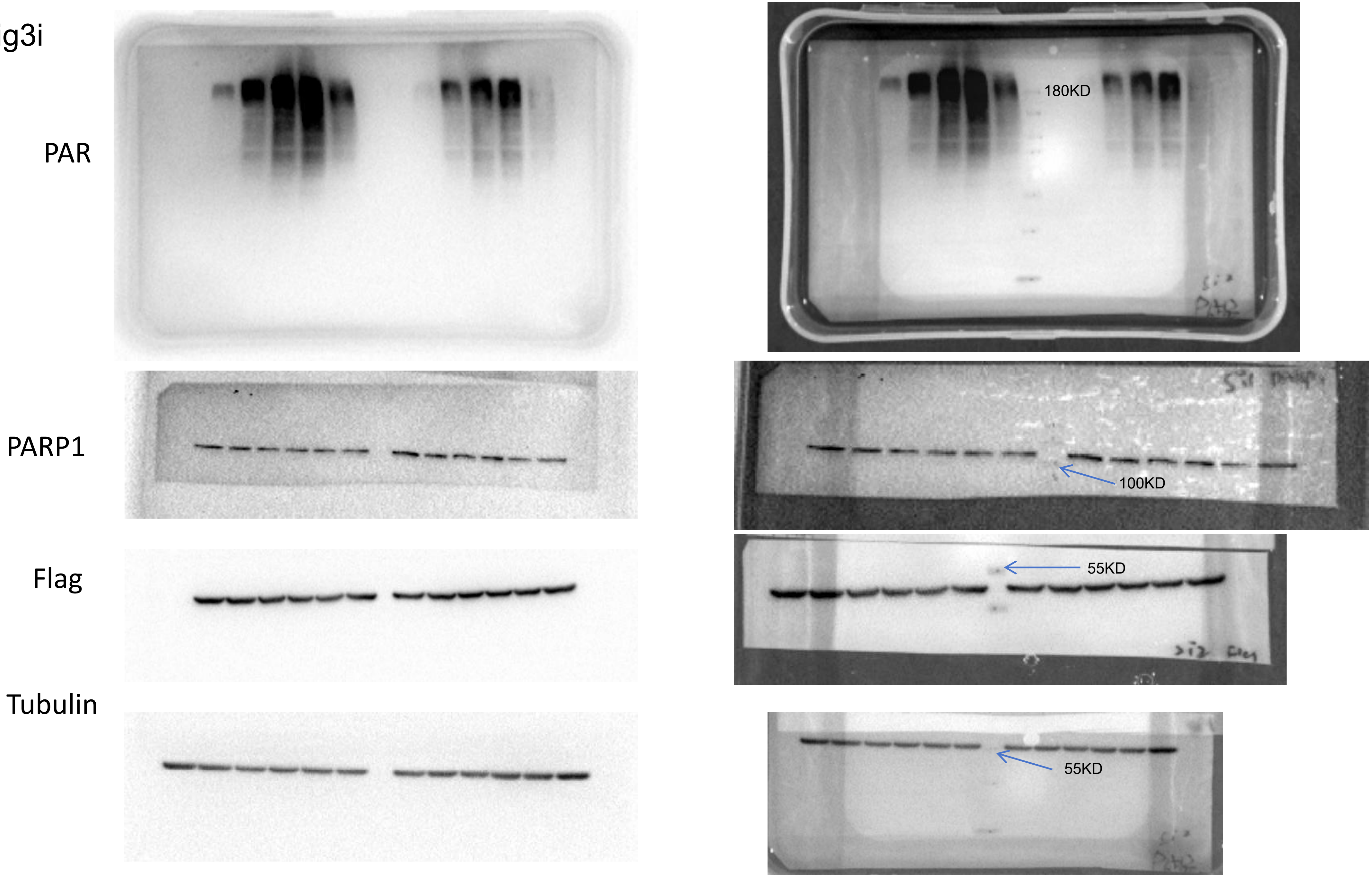

Fig4c

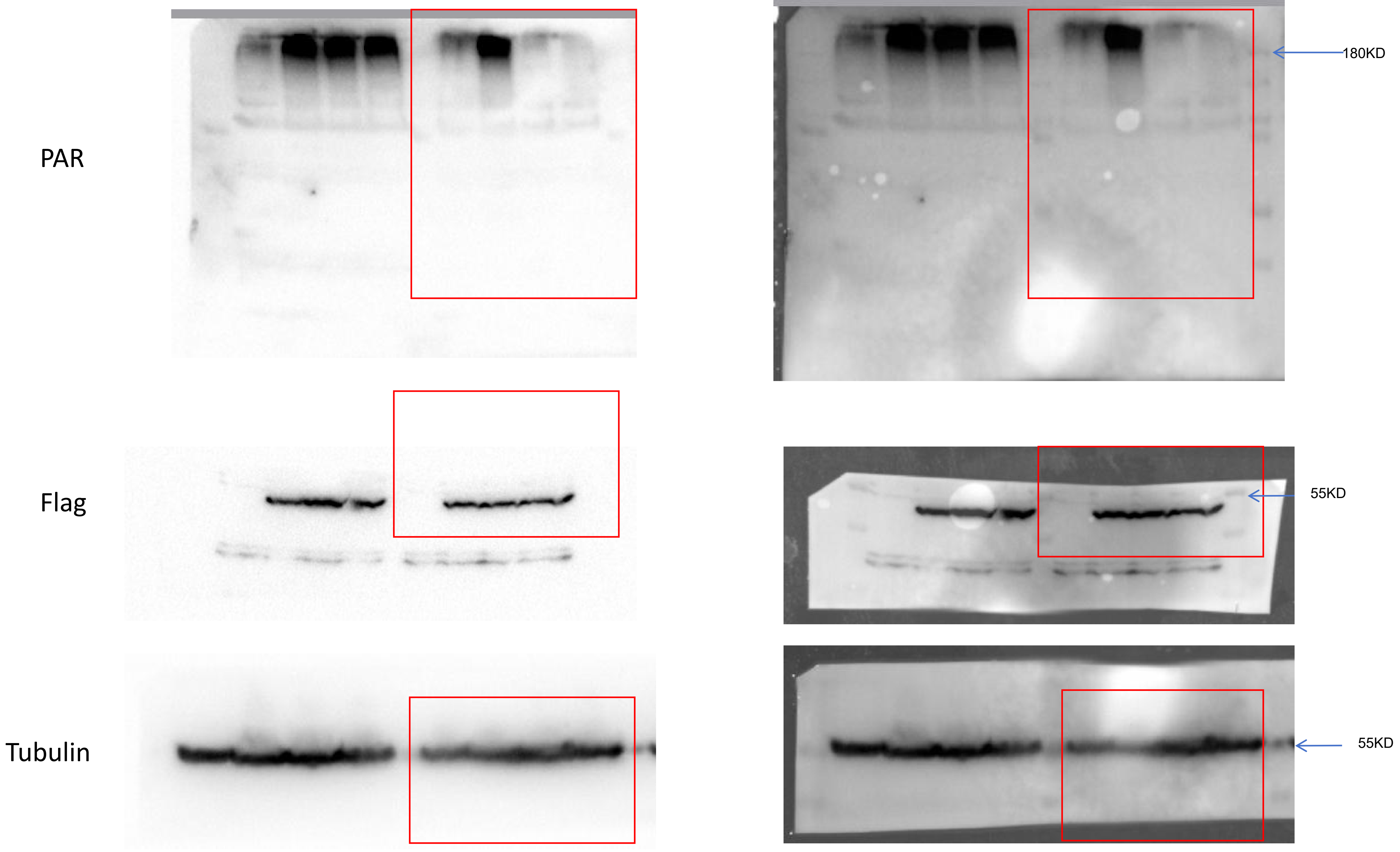

Fig4d

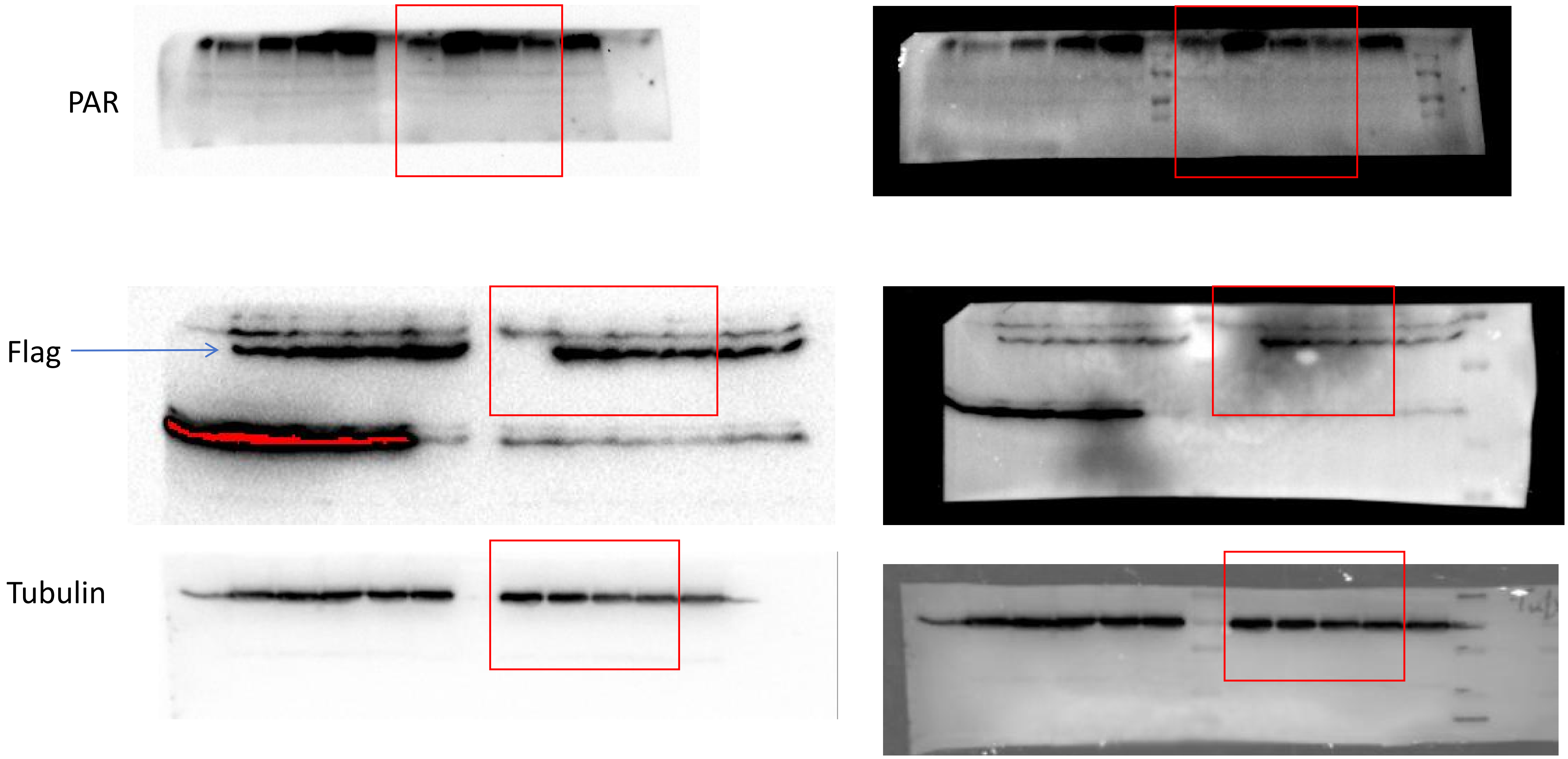

Fig4e

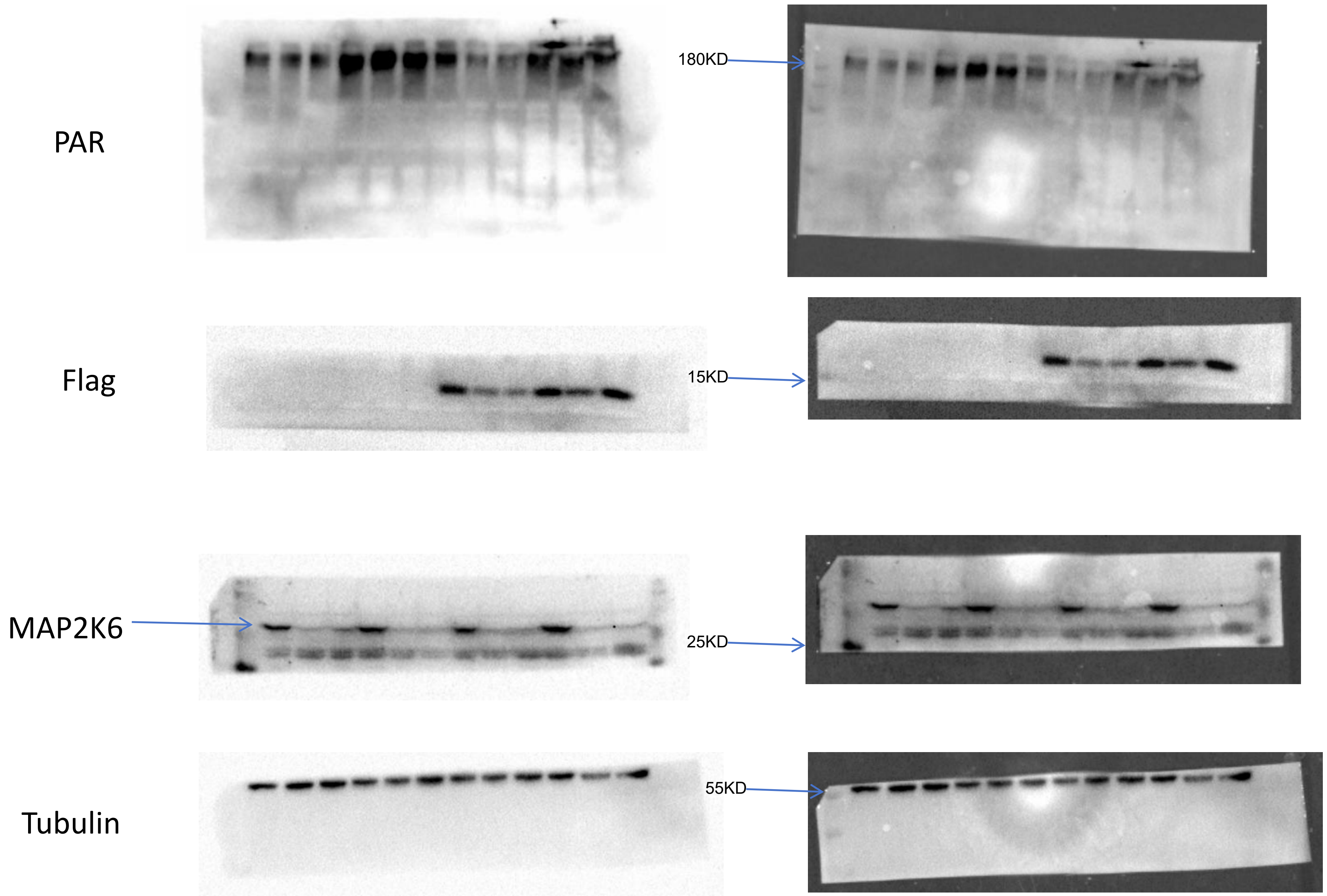

Extended Data Fig.2e

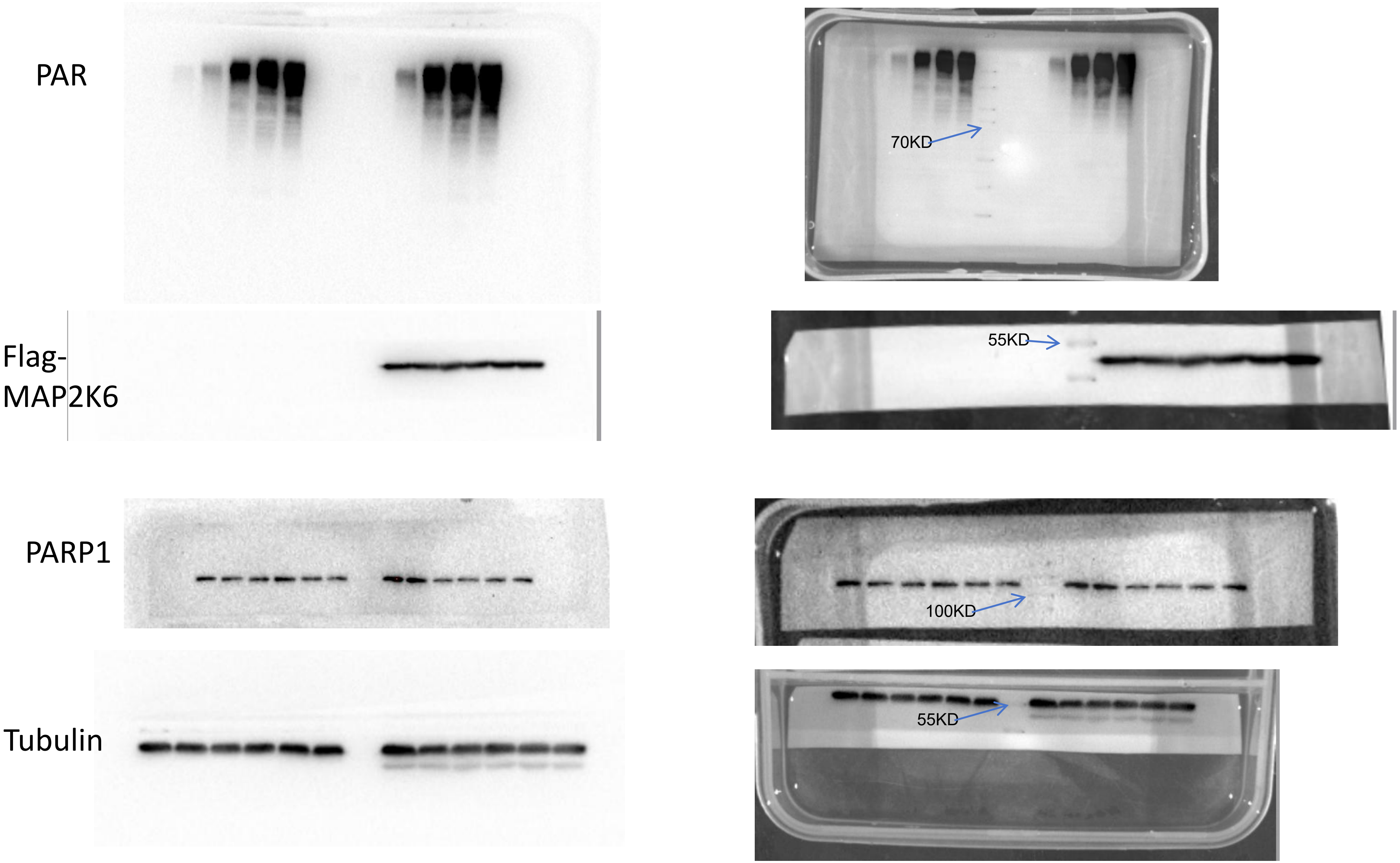

Extended Data Fig.2f

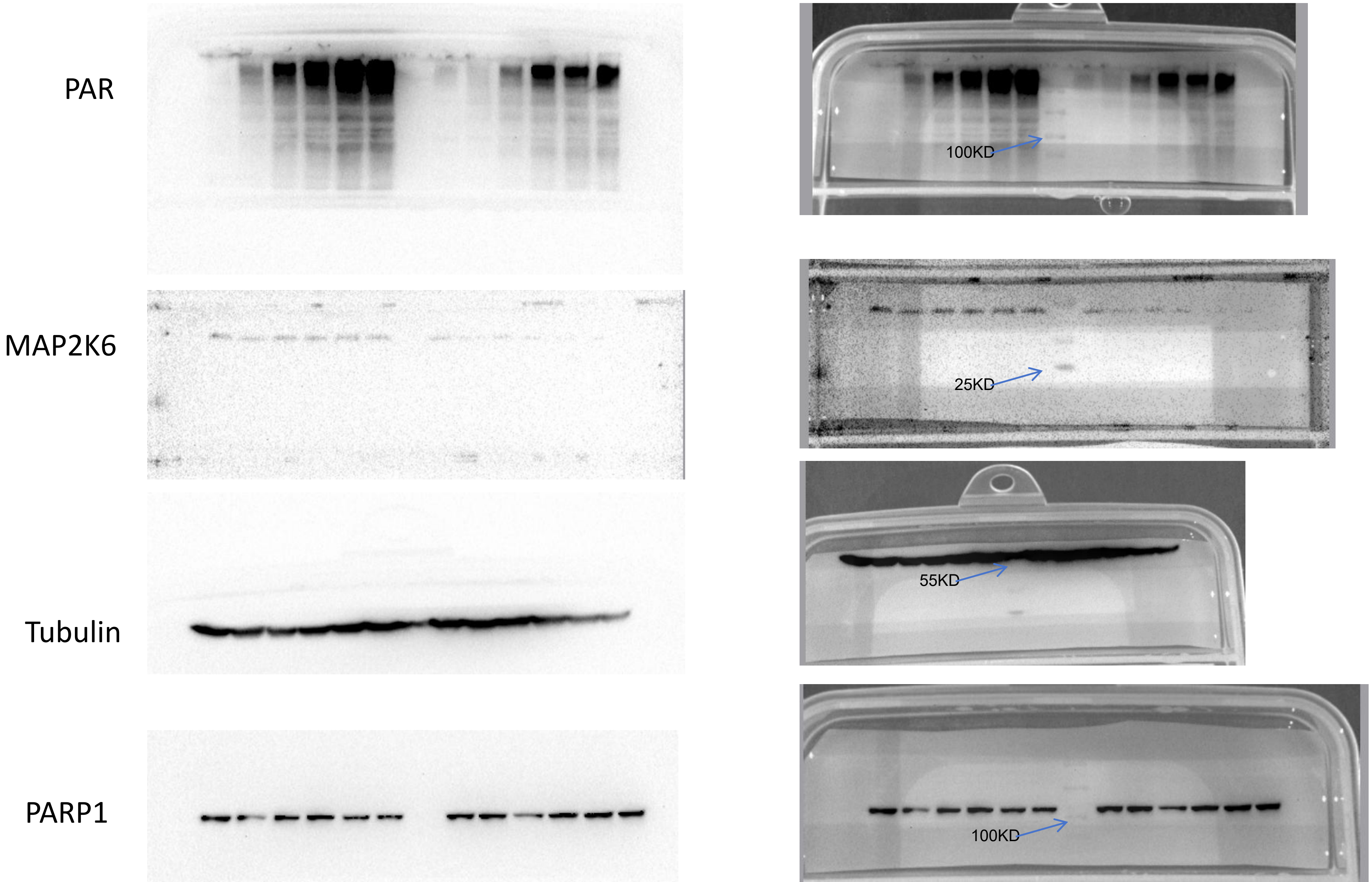

Extended Data Fig.2g

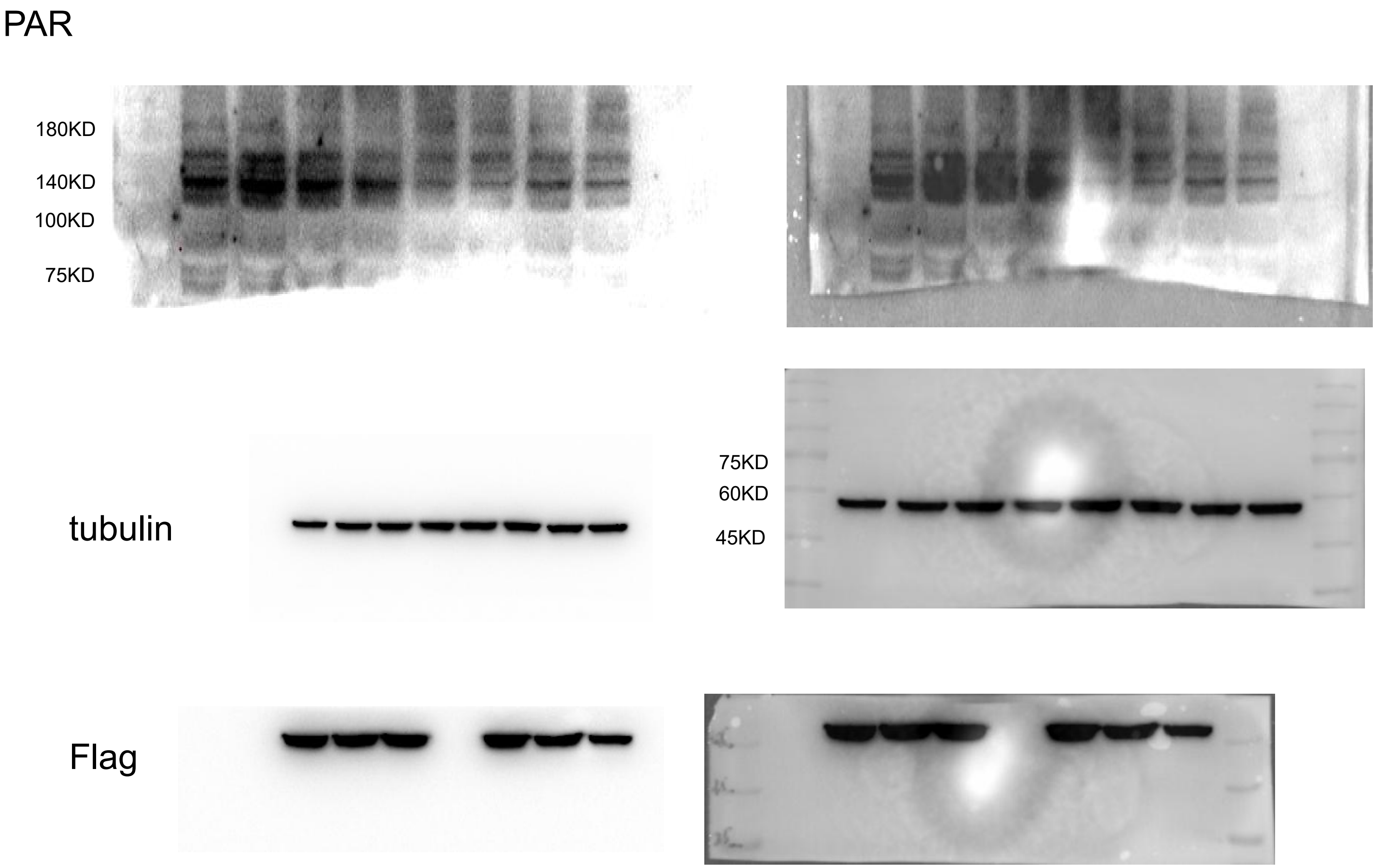

Extended Data Fig.2h

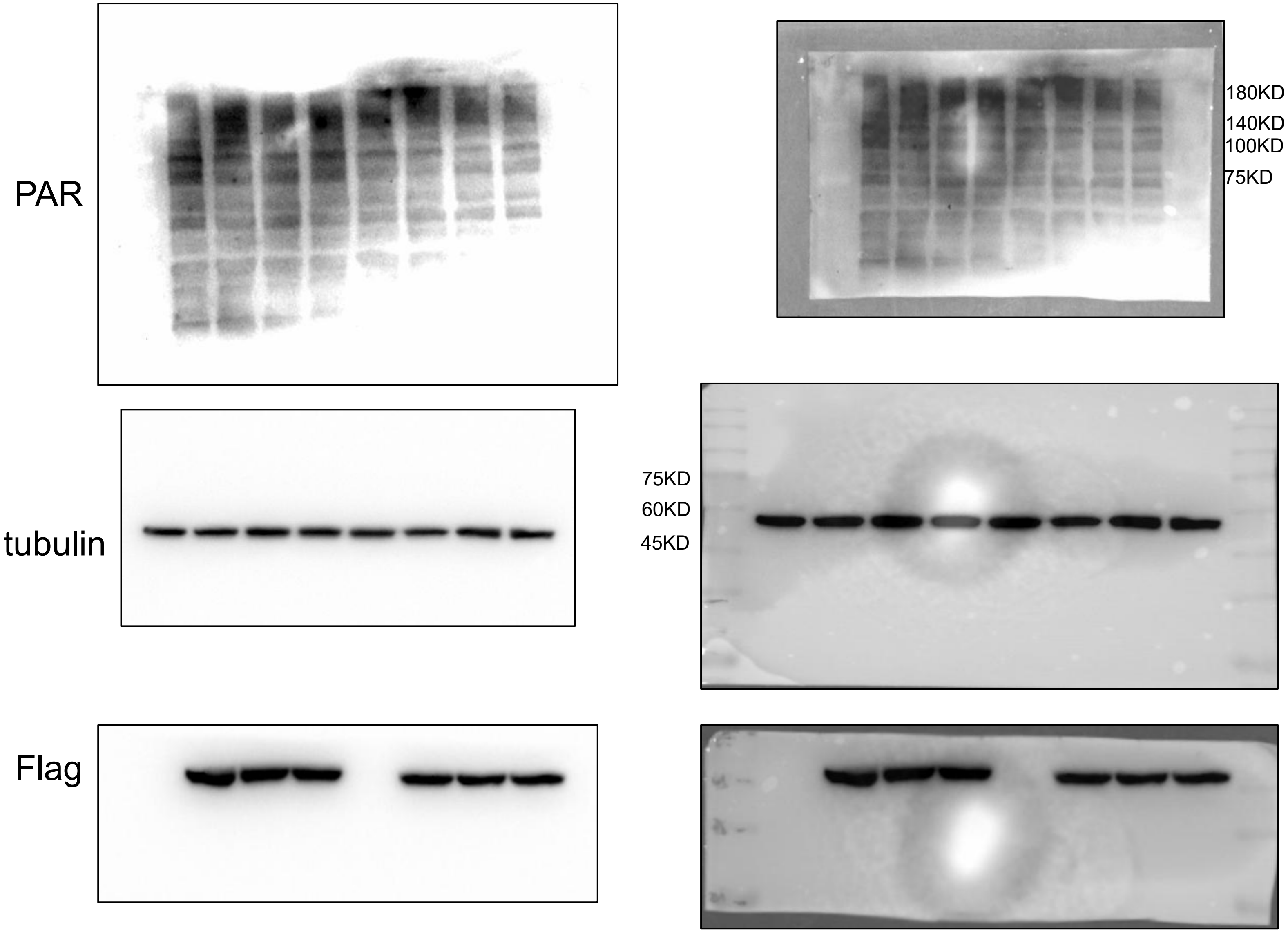

Extended Data Fig.2i

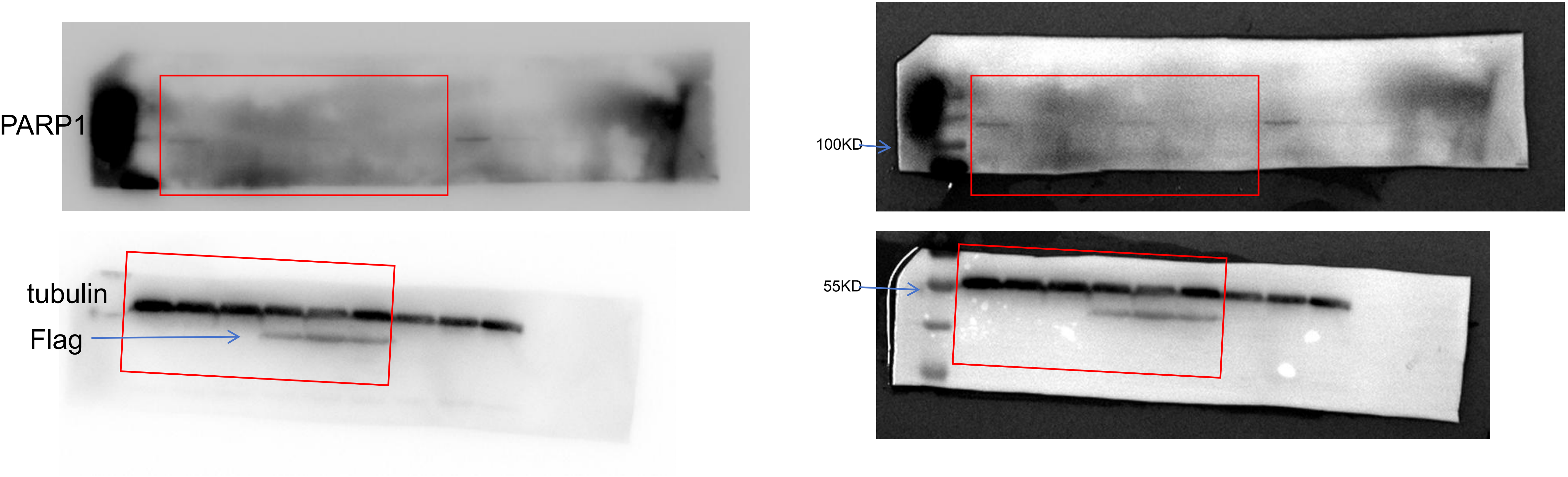

Extended Data Fig.2k

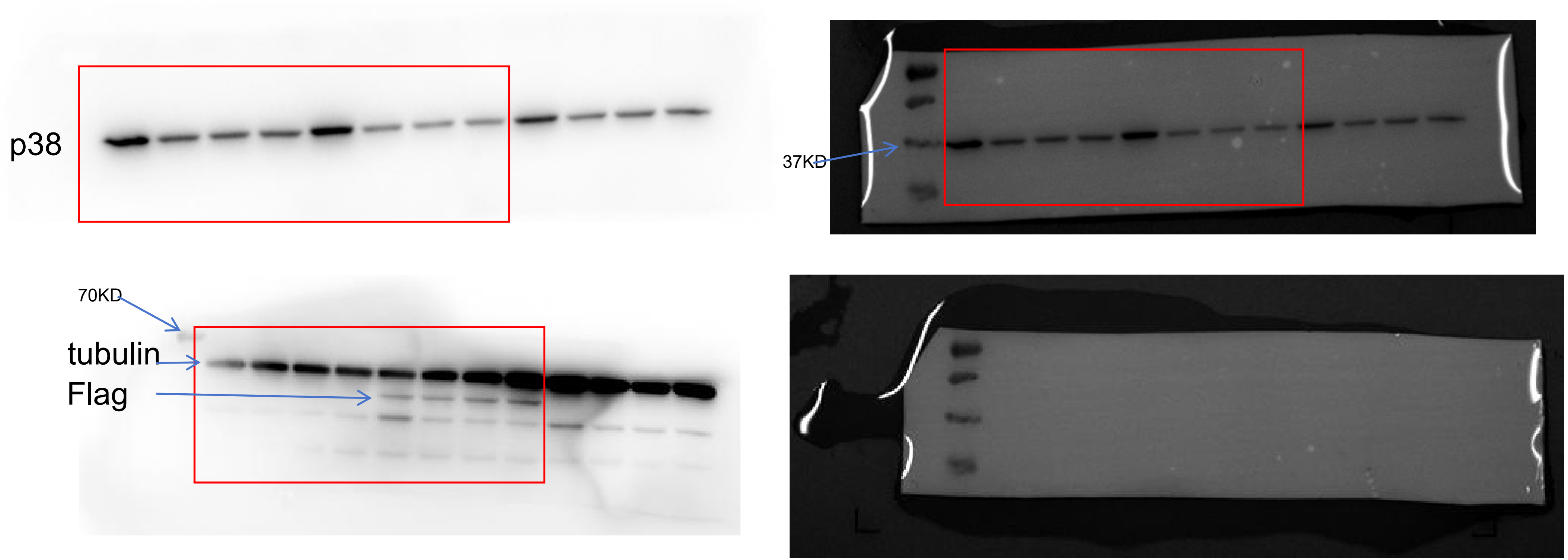

Extended Data Fig.2l

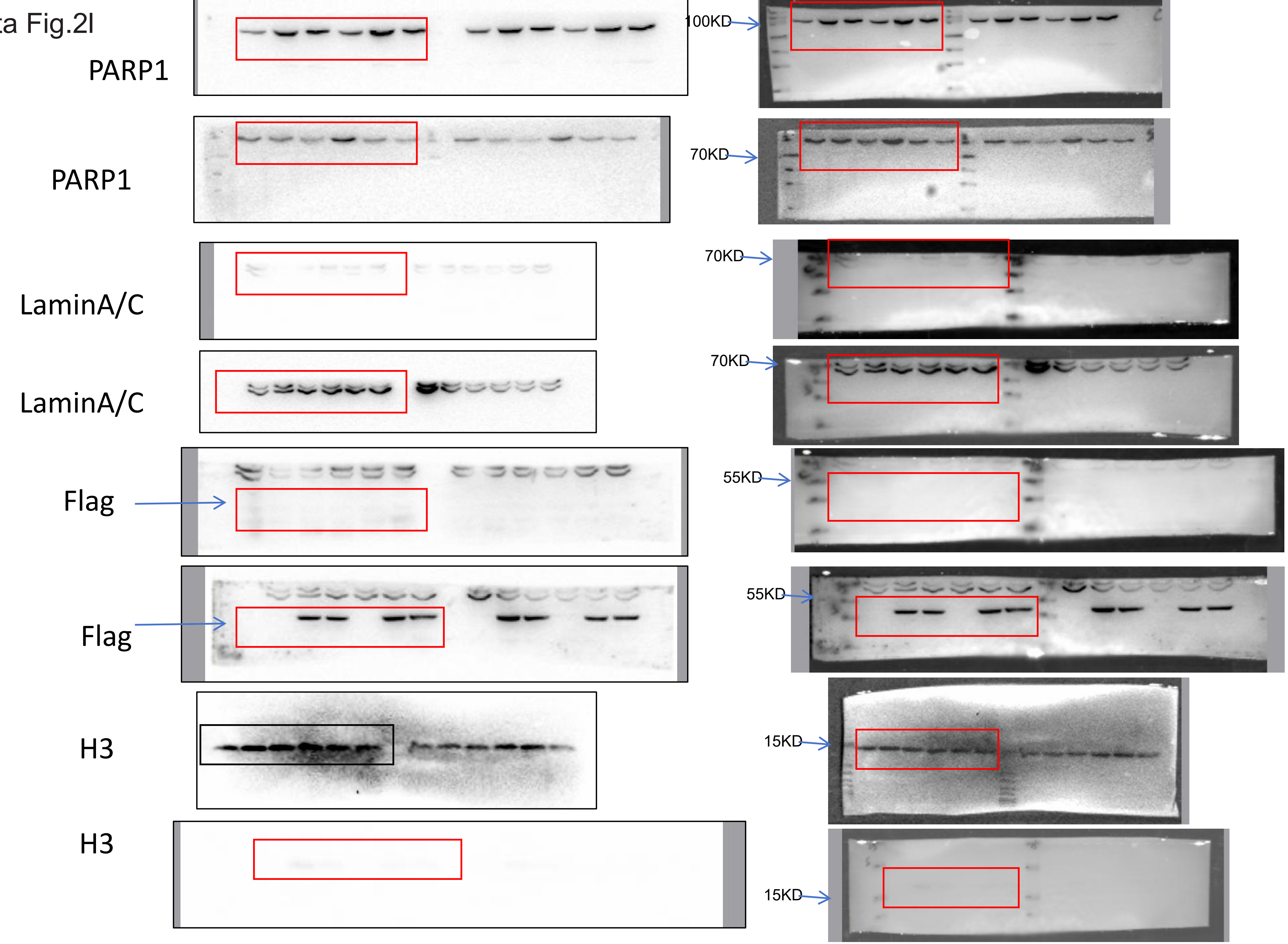

Extended Data Fig3g

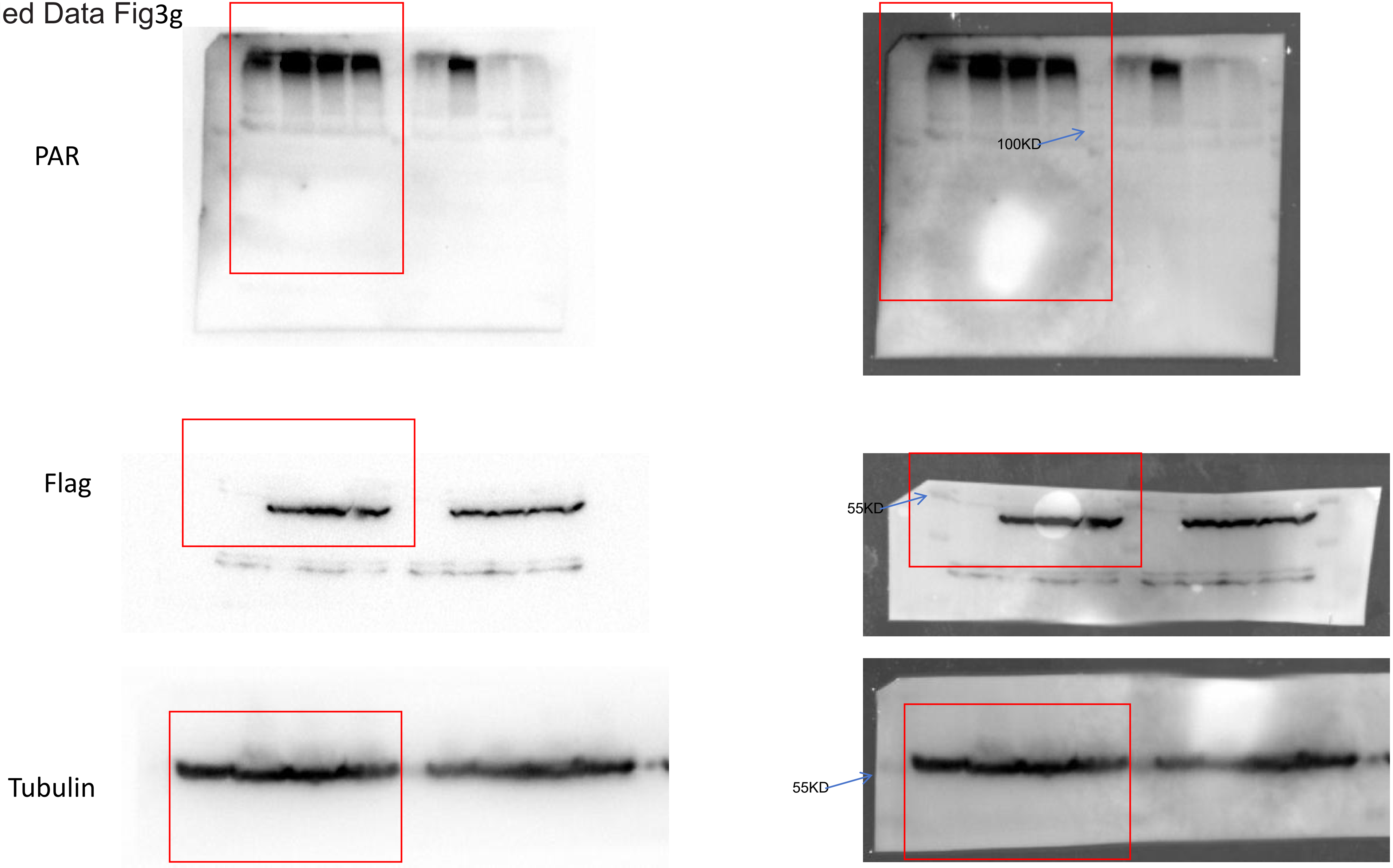

Supplement: Supplementary file 1 [file 41419_2026_9104_MOESM9_ESM.pdf]
