# Supplementary material for: MAP2K6 reprograms NAD+ metabolism via the HMGA1–NAMPT axis to sensitize ovarian cancer cells to PARP inhibition
Source: Cell Death Dis. 2026 Jul 17;17(1):880. doi: 10.1038/s41419-026-09104-2 (PMC13658224; doi:10.1038/s41419-026-09104-2)
Supplement: Supplementary file 2 [file 41419_2026_9104_MOESM1_ESM.docx]

**Extended Figures**

**Figure S1. MAP2K6 is a predictive marker of PARP inhibitor sensitivity in ovarian cancer**

a. Pearson correlation matrix with heatmap of proteomic profiles of 11 ovarian cancer cell lines, each with three biological replicates.

**b.**The accumulation of gene products identified in each sample.

**c.** Barplot showing the gene products identified in each group.

**d.** Top 10 negatively correlated genes identified in Fig 1d spearman correlation analysis. Genes with spearman correlation < -0.5 and p-value <= 0.05 were defined as olaparib sensitive related genes.

**e-f.** Forest Plot of the univariate (e) and multivariate (f) Cox proportional hazards analysis of PFS (Progression Free Survival) for PARPi in the testing cohort TableS1A.

**Figure S2. MAP2K6 modulates PARylation and olaparib response in ovarian cancer cells**

**a-b.** CCK8 cell viability of A2780 cells transiently transfected siCtrl or siMAP2K6 following treatment with 5 μM olaparib for 28 h measured by CCK-8 assay (a). Relative MAP2K6 mRNA levels in corresponding cells (b). (two-tailed unpaired Student’s t tests. ***p < 0.001; error bar = mean ±SEM, n = 6).

**c-d.** CCK8 cell viability of OVCAR3 and OVCA429 cell lines expressing siCtrl or MAP2K6-targeted siRNA (siMAP2K6) treated with 10 μM or 20 μM olaparib for 48 h (two-tailed unpaired Student’s t tests. *p < 0.0, **p < 0.01, ***p < 0.001, error bar = mean ±SEM, n = 6).

**e.** Western blot of PARylation level in OAW42 cell lines stably expressing Flag or Flag-MAP2K6 treated with 2 mM H_2_O_2_ for the indicated time point. Quantification of PARylation normalized to tubulin is shown on the right.

**f.** Western blot analysis of PARylation in A2780 cell lines transiently transfected siNC or si1-MAP2K6 treated with 2 mM H_2_O_2_ for the indicated time. Quantification of PARylation normalized to tubulin is shown on the right.

**g-h.** Western blot of PARylation level in OAW42 (g) and C13 (h) cell lines stably expressing Flag or Flag-MAP2K6 treated with p38 inhibitors (20μM Adezmapimod or 10μM SD202190) and 50μM olaparib 24h as indicated.

**i.** Western blot analysis of PARP1 and Flag-MAP2K6 expression levels in the indicated cells.

**j.** CCK8 cell viability of OAW42 cells stably expressing vector or MAP2K6 with PARP1 knockdown treated with olaparib (50 μM) for 48 h (two-tailed unpaired Student’s t tests. ***p < 0.001; error bar = mean ±SEM, n = 6).

**k.** CCK8 cell viability of OAW42 cells stably expressing vector or MAP2K6 with p38 knockdown treated with 100 μM olaparib for 48 h. Western blots of p38 levels in OAW42 cells expressing Flag or Flag-MAP2K6 with p38 knockdown are shown on the right (two-tailed unpaired Student’s t tests. ***p < 0.001; error bar = mean ±SEM, n = 6).

**l.** Western blot analysis of chromatin and and nuclear soluble fractions prepared from OAW42 cells stably expressing Flag or Flag-MAP2K6 without drug or pre-treated with p38 inhibitors 10μM SD202190 and 50μM olaparib 4 h as indicated. Blots were probed with indicated antibodies. Histone H3 and LaminA/C were used as positive markers for chromatin and nuclear soluble fractions, respectively.

**m.** PARP1 protein abundance identified in TFRE proteomics across 11 ovarian cancer cell lines (error bar = mean ±SEM, n = 3).

**n.** Pearson's correlation analysis between PARP1 protein abundance identified in TFRE proteomics and MAP2K6 protein level in profiling. P-Value of pearson's correlation was calculated by R function cor.test.

**o.** Dotplot showing association of protein abundance identified in TFRE and correlation between protein level and PARPi IC50.

**Figure S3. MAP2K6 promotes NAD+ synthesis and PARylation through the NAMPT pathway**

**a.b.** Measurement of cellular NAD+ using the NAD/NADH-Glo™ Assay in C13 (a) and OAW42 (b) cell lines stably expressing Flag or Flag-MAP2K6 (two-tailed unpaired Student’s t tests. *p < 0.05; error bar = mean ±SEM, n = 3).

**c.** Measurement of cellular NAD+ using the NAD/NADH-Glo™ Assay in C13 cell lines stably expressing Flag or Flag-MAP2K6 pre-treated with 20μM Adezmapimod or 10μM SD202190 as indicated.( two-tailed unpaired Student’s t tests. *p < 0.05;**p < 0.01;***p < 0.001; error bar = mean±SEM, n = 3).

**d-e.** Relative NAMPT mRNA expression in OAW42(d) and C13(e) cells stably expressing Flag or Flag-MAP2K6 treated with 20μM Adezmapimod or 10μM SD202190 24h as indicated.( two-tailed unpaired Student’s t tests. *p < 0.05;***p < 0.001; error bar = mean±SEM, n = 3).

**f.** Relative NAMPT mRNA expression in C13 cell lines stably expressing Flag-MAP2K6 following NAMPT knockdown (error bar = mean ±SEM, n = 3).

**g.** Western blots of PARylation level in OAW42 cell lines stably expressing Flag or Flag-MAP2K6 with NAMPT knockdown.

**h.** Relative NAMPT mRNA expression in OAW42 cell lines stably expressing Flag-MAP2K6 with NAMPT knockdown (two-tailed unpaired Student’s t tests. *p < 0.05; error bar = mean ±SEM, n = 3).

**Figure S4. MAP2K6 signaling enhances NAMPT transcription via HMGA1**

**a.** Principal component analysis (PCA) of phosphorylated proteomes of C13 stably expressing Flag and Flag-MAP2K6 cells.

**b.** Chromatin immunoprecipitation (ChIP) assays using anti-Flag antibodies in 293FT cells expressing the indicated plasmids and treated with 20μM SD202190 24 h as indicated. Pulled-down DNA was analyzed by qPCR using primers targeting the NAMPT enhancer. (two-tailed unpaired Student’s t tests, ***p < 0.001; error bar = mean±SEM, n = 4).

**c-d.** Relative HMGA1 mRNA expression in C13 (c) or OAW42 (d) cells stably expressing Flag-MAP2K6 with HMGA1 knockdown (error bar = mean ±SEM, n = 3).

**e.** CCK8 cell viability of C13 cells stably expressing vector or MAP2K6 with HMGA1 knocked down or re-expression of siHMGA1#1 resistant HMGA1 mutants as indicated, treated with olaparib (50 μM) for 48 h (two-tailed unpaired Student’s t tests. *p < 0.05; ***p < 0.001; error bar = mean ±SEM, n = 6).

**Figure S5. MAP2K6 enhances olaparib antitumor efficacy in vivo**

**a.** Heatmap showing the indicated protein expression patterns in a proteomic cohort across 183 ovarian cancer samples as reported in reference^1^.

**b.** Pearson’s correlation analysis of MAP2K6 and NAMPT protein abundance across 183 tumor samples. p value calculated using the cor.test function in R.

**c.** Pearson’s correlation analysis of MAP2K6 and NAMPT protein abundance in BRCA WT patients in the study by Guo et al., 2024 (Nature Communications, PMID: 39085232)^2^.

**Reference**

1.	Tan R, Wen M, Yang W, Zhan D, Zheng N, Liu M*, et al.* Integrated proteomics and scRNA-seq analyses of ovarian cancer reveal molecular subtype-associated cell landscapes and immunotherapy targets. *British journal of cancer* 2025, **132**(1)**:** 111-125.

2.	Qian L, Zhu J, Xue Z, Zhou Y, Xiang N, Xu H*, et al.* Proteomic landscape of epithelial ovarian cancer. *Nature communications* 2024, **15**(1)**:** 6462.
